# Complete Phenotypic Recovery of an Alzheimer's Disease Model by a Quinone-Tryptophan Hybrid Aggregation Inhibitor

**DOI:** 10.1371/journal.pone.0011101

**Published:** 2010-06-14

**Authors:** Roni Scherzer-Attali, Riccardo Pellarin, Marino Convertino, Anat Frydman-Marom, Nirit Egoz-Matia, Sivan Peled, Michal Levy-Sakin, Deborah E. Shalev, Amedeo Caflisch, Ehud Gazit, Daniel Segal

**Affiliations:** 1 Department of Molecular Microbiology and Biotechnology, Tel-Aviv University, Tel-Aviv, Israel; 2 Department of Biochemistry, University of Zurich, Zurich, Switzerland; 3 Wolfson Centre for Applied Structural Biology, Hebrew University of Jerusalem, Jerusalem, Israel; University of Canterbury, New Zealand

## Abstract

The rational design of amyloid oligomer inhibitors is yet an unmet drug development need. Previous studies have identified the role of tryptophan in amyloid recognition, association and inhibition. Furthermore, tryptophan was ranked as the residue with highest amyloidogenic propensity. Other studies have demonstrated that quinones, specifically anthraquinones, can serve as aggregation inhibitors probably due to the dipole interaction of the quinonic ring with aromatic recognition sites within the amyloidogenic proteins. Here, using *in vitro*, *in vivo* and *in silico* tools we describe the synthesis and functional characterization of a rationally designed inhibitor of the Alzheimer's disease-associated β-amyloid. This compound, 1,4-naphthoquinon-2-yl-L-tryptophan (NQTrp), combines the recognition capacities of both quinone and tryptophan moieties and completely inhibited Aβ oligomerization and fibrillization, as well as the cytotoxic effect of Aβ oligomers towards cultured neuronal cell line. Furthermore, when fed to transgenic Alzheimer's disease Drosophila model it prolonged their life span and completely abolished their defective locomotion. Analysis of the brains of these flies showed a significant reduction in oligomeric species of Aβ while immuno-staining of the 3^rd^ instar larval brains showed a significant reduction in Aβ accumulation. Computational studies, as well as NMR and CD spectroscopy provide mechanistic insight into the activity of the compound which is most likely mediated by clamping of the aromatic recognition interface in the central segment of Aβ. Our results demonstrate that interfering with the aromatic core of amyloidogenic peptides is a promising approach for inhibiting various pathogenic species associated with amyloidogenic diseases. The compound NQTrp can serve as a lead for developing a new class of disease modifying drugs for Alzheimer's disease.

## Introduction

Alzheimer's disease (AD), a progressive neurodegenerative disorder for which there is no cure or effective treatment, is the leading cause of dementia in aged humans. Symptoms include memory loss, confusion, impaired judgment, personality changes, disorientation and loss of language skills [1], [2]. The major neuropathological changes in the brains of AD patients include neuronal death, particularly in regions related to memory and cognition and the presence of intra- and extra-cellular abnormal protein aggregates [3], [4] known as neurofibrillary tangles and amyloid plaques, respectively. In the past several years a large body of evidence has established a pathological role for β-amyloid polypeptide (Aβ) in AD [5]–[10]. Accumulating evidence indicate a fundamental role of the early soluble oligomeric species of Aβ, rather than the mature fibrillar species, in the pathogenesis of AD [11]–[15]. Yet, the molecular mechanism underlying the assembly of the different Aβ species is not fully understood. However, since these structures self-assemble, from monomers to higher oligomeric or fibrillar structures in a highly ordered and efficient manner, it is likely that specific recognition elements mediate the process.

We and others have identified a central role of aromatic residues in formation and stabilization of amyloid structures [16]–[19]. This notion has gained direct evidence by high-resolution structural studies [20], [21], theoretical analysis and molecular dynamics simulations [22]–[25]. Among the aromatic moieties, tryptophan was ranked as the residue with highest amyloidogenic potential by Dobson and co-workers [26] and an un-biased analysis, using peptide array technology, has clearly indicated a significantly higher affinity of tryptophan-modified recognition module in the molecular association of the islet amyloid polypeptide [27]. Indeed, as expected from these findings, several small aromatic molecules such as polyphenols [28]–[30] and small aromatic peptides [31] were shown to inhibit the aggregation of several amyloidogenic peptides. Furthermore, we have shown significant inhibition *in vitro* of the Aβ polypeptide by indole derivatives [32]. Moreover, we have recently demonstrated efficient inhibition of Aβ oligomerization by a short D-tryptophan-Aib dipeptide both *in vitro* and *in vivo* [31], further underscoring the important role of tryptophan in the binding and inhibition of Aβ. These findings have led to the suggestion that targeting of aromatic recognition interfaces by tryptophan could be a useful strategy for anti-amyloid formation.

Quinones have long been known to act as inhibitors of various metabolic pathways in the cell, to have anti-bacterial, anti-viral, and also anti-cancer activities [33], [34]. Several quinones have been shown to be effective inhibitors of the aggregation of several amyloidogenic proteins. For example, *p*-benzoquinone was reported to reduce the toxicity of islet amyloid peptide aggregates [35] and inhibit amyloid fibril formation by hen egg-white lysozymes [36]. Likewise, anthraquinones were demonstrated to be effective inhibitors of Tau protein aggregation [37]. Recently, 1,2-naphthoquinone was shown to effectively inhibit Aβ_42_ oligomerization *in vitro* [38]. It appears that the asymmetric dipole of the quinonic ring plays a central role in the interaction between the molecule and the amyloidogenic peptides. The interactions at the basis of the anti-amyloid activity of anthraquinone (a tri-cyclic quinone) were recently shown to be the hydrogen bonds, the aromatic contacts and, moreover, the ability to establish a favorable interaction between the central electron-poor quinonic ring and the electron-rich peptidic carbonyls [39].

Here we sought to combine the strong interaction and recognition between tryptophan and the Aβ peptide with the documented inhibitory capability of quinones towards Aβ assembly. To that end we examined the effect of 12 different hybrid molecules, consisting of a naphthoquinone and different linked residues, towards Aβ oligomerization and fibrillization. Among the compounds tested the hybrid 1,4-naphthoquinon-2-yl-L-tryptophan (termed hereafter NQTrp) [40] was found to be the most effective.

We hypothesize that intermolecular alignment of the phenylalanine (at position 19 or 20 of the Aβ sequence) intercalated between the flat electron-deficient naphthoquinone moiety and the high electron-dense indole ring of the tryptophan, would lead to formation of a near face-to-face stable complex. Due to near face-to-face and edge-to-face geometry accompanied by sterical hindrance, the intermolecular complex of the aromatic elements effectively prevents Aβ assembly. Structural analysis supports this proposed mode of action of NQTrp. *In vivo* assays demonstrate that Aβ inhibition is accompanied by significant amelioration of AD-engendered symptoms.

## Results

Twelve naphthoquinone hybrid molecules were screened for their ability to inhibit formation of Aβ oligomers and fibrils *in vitro* [Figure S1, Table S1]. All twelve molecules included a 1,4-naphthoquinone, but with different residues linked to it, some aromatic and some not. All hybrid molecules were analyzed both in the oligomer inhibition assay and ThT fibril inhibition assay described below for NQTrp, followed by TEM analysis (not shown). Results of all hybrids are summed up in Table S1. They show that NQTrp had strongest inhibition activity, towards the formation of both Aβ oligomers and fibrils. It is also apparent that both the D isomer of NQTrp (compound IID in Table S2) and the indole derivative (compound III) are good inhibitors. These results strongly suggest that the linking between 1,4-naphthoquinone and a molecule containing an indole ring is crucial for optimal inhibition.

### Inhibition of toxic Aβ oligomer species

**Figure 1:**
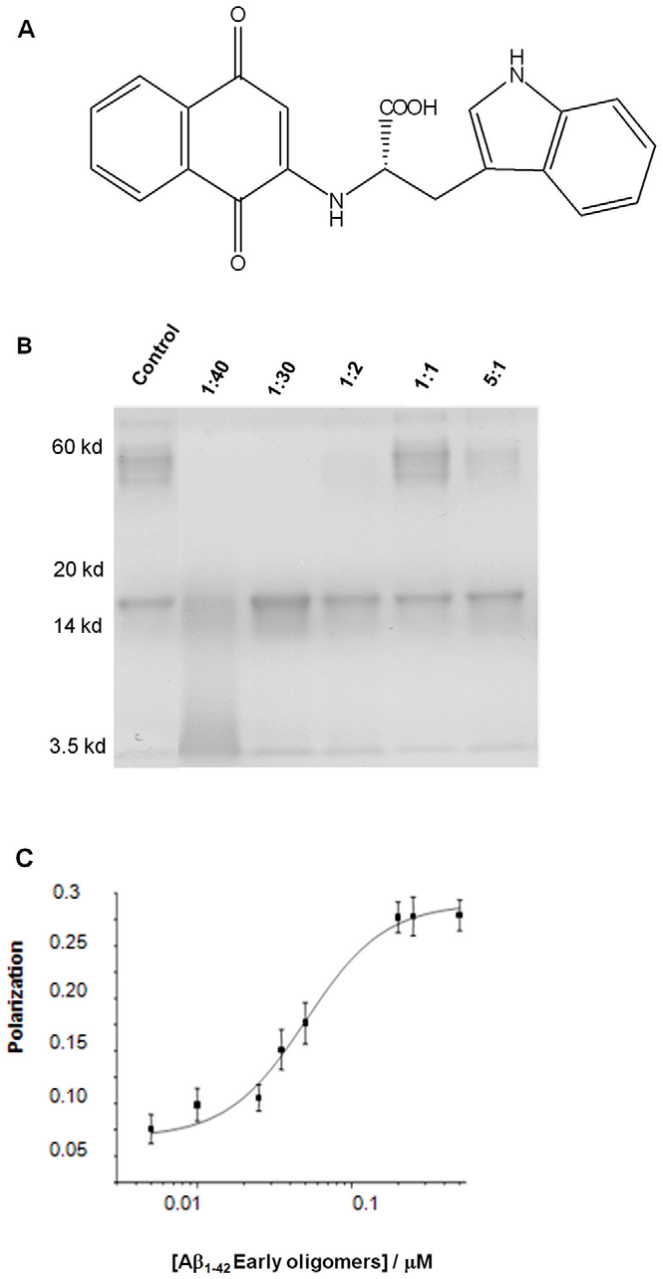
Inhibition of Aβ oligomer formation *in vitro*. **A**. Structure of 1,4-naphthoquinon-2-yl-L-tryptophan (NQTrp). **B**. Determination of the dose-dependent effect of NQTrp on soluble oligomer formation. Soluble oligomers were prepared according to the method of Barghorn *et al.* [15] with and without increasing concentration of NQTrp. Aβ concentration was set at 133 µM. Molar ratios of Aβ∶NQTrp are indicated. The control is Aβ only. **C**. The affinity of NQTrp towards early oligomers was determined using fluorescence anisotropy.

The effect of NQTrp on the ability of early non-toxic intermediate Aβ oligomers (∼18 kDa) to further grow into the toxic dodecameric oligomer assemblies (∼56 kDa) was analyzed using the protocol established by Hillen and coworkers [15]. This protocol results in the formation of SDS-stable oligomers that display toxic effects on the long-term potentiation of cultured neural cells [15]. For example, to evaluate the effect of NQTrp (Figure 1A) on the transformation of the Aβ into the toxic assemblies, the inhibitor was incubated with Aβ_1–42_ at increasing molar ratios, and the reaction mixtures were resolved on SDS-PAGE (Figure 1B). The results reveal dose-dependent inhibition, by NQTrp, of the ability of Aβ to assemble into toxic oligomers (∼56 kDa), inhibition was apparent at a low 5∶1 (Aβ_1–42_∶NQTrp); however the inhibition profile is non linear. The decreased inhibition effect at mid-range molar rations such as at a 1∶1 ratio may be due to a competing homomolecular noncovalent interaction as observed for various other small molecular inhibitors such as indole moieties and small peptides. The inhibitor appears to stabilize the non-toxic early oligomers and inhibit their further growth into toxic species. Complete inhibition was seen only at molar excess of NQTrp.

### Characterization of the interaction between NQTrp and Aβ

The affinity of NQTrp towards the early Aβ_1–42_ assemblies was demonstrated using fluorescence anisotropy assay, taking advantage of the intrinsic fluorescence of the Trp-substituted quinone and its relatively small size as compared to the Aβ oligomers. Increasing amounts of early assemblies of Aβ were titrated into a solution of NQTrp and anisotropy was determined (Figure 1C). The affinity constant of NQTrp was estimated to be 90 nM.

### Inhibition of amyloid fibril formation by NQTrp

**Figure 2:**
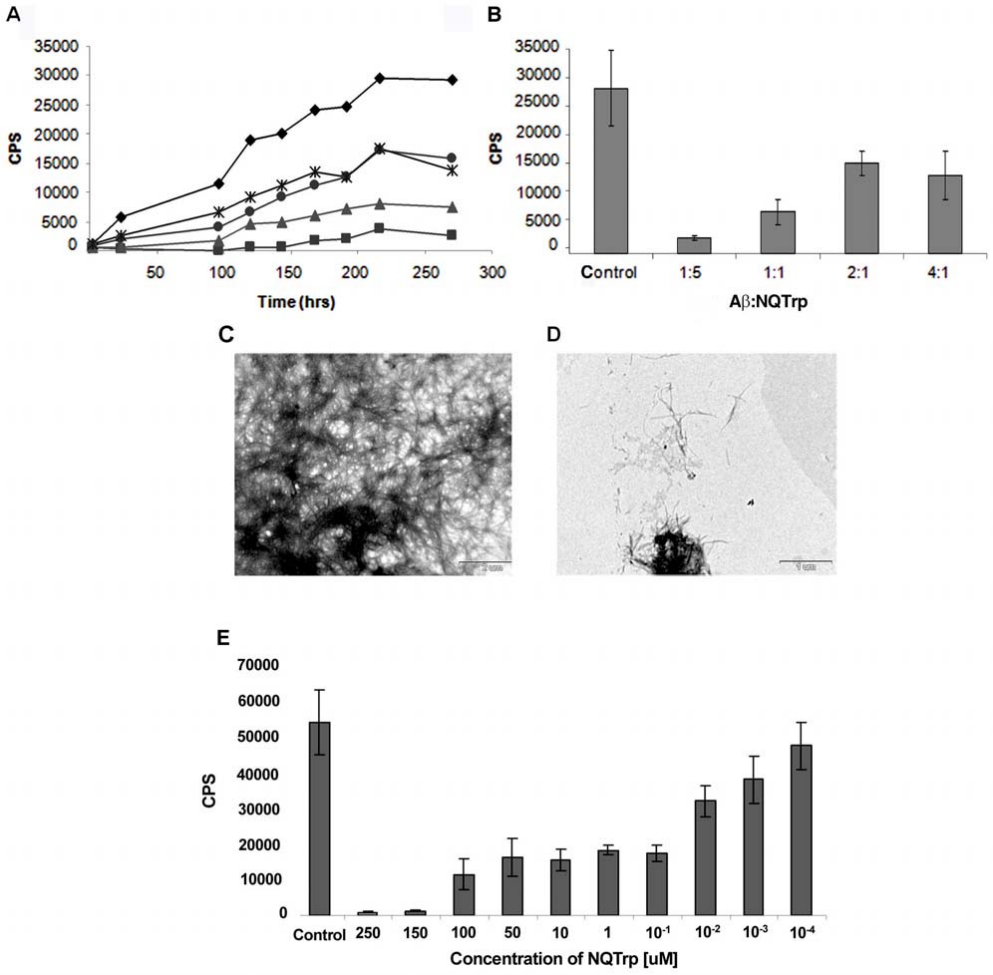
Inhibition of Aβ fibril formation *in vitro*. **A**. Dose dependent kinetic analysis of the inhibition of NQTrp towards fibril formation of Aβ_1–42_ over the course of 270 hours. Aβ concentration was set to 5µM. Concentrations are expressed as Aβ∶quinone molar ratio: Control - Aβ_1–42_ only (♦), 1∶5 (▪), 1∶1 (▴), 2∶1 (•), 4∶1 (_*_). (CPS = Counts Per Second) **B**. Endpoint of ThT analysis after 270 hours. **C–D**. Transmission Electron Microscope images taken from ThT analysis after 270 hours. Aβ_1–42_ alone (**C**), Aβ_1–42_ with NQTrp (1∶5) (**D**). E. Dose dependent inhibition by NQTrp of the fibrillization of Aβ_1–42_ (ThT assay). Concentration of Aβ_1–42_ was set to 5uM. Control - Aβ_1–42_ alone. An IC_50_ of 50nM was calculated.

The relative contribution of Aβ fibrils versus oligomers to the pathogenesis of AD has not been completely resolved [41]. We therefore wanted to discern whether or not NQTrp also inhibits the formation of mature β-amyloid fibrils. To that end we used the Thioflavin-T (ThT) binding assay, which provides a quantitative measure of amyloid fibril formation. Aβ_1–40_ was allowed to form amyloid fibrils either in the absence or in the presence of increasing concentrations of NQTrp (Figure 2A). The process of fibrillization was followed for several days until a plateau was reached and its kinetics was measured. The formation of Aβ fibrils was significantly reduced in the presence of the inhibitor, even at low molar ratios of 4∶1 (Aβ_1–40_∶NQTrp). This is especially evident after 270 hours (Figure 2B). A similar experiment using Aβ_1–42_ resulted in IC_50_ of 50 nM (Figure 2E). These results clearly indicate that the NQTrp is an effective inhibitor of Aβ fibril formation.

The morphology of the Aβ fibrils formed during the course of fibrillization was compared, in the presence and in the absence of NQTrp, using transmission electron microscopy (TEM). Samples were taken from the amyloid fibril formation experiment after 7 days of incubation. The fibrils formed by Aβ alone were large, broad and ribbon-like (Figure 2C). The samples containing Aβ and NQTrp showed drastic reduction of fibrils. The few fibrils that formed in the presence of the inhibitor were much thinner and shorter (Figure 2D). This strongly correlated with the values observed in the amyloid fibril formation experiment.

### NMR analysis of the interaction of NQTrp with Aβ

To characterize the precise interaction between NQTrp and Aβ, NMR analysis was conducted. NQTrp was incubated with a truncated fragment of Aβ, Aβ_12–28_, which is a less-aggregative fragment, commonly used to avoid complications of oligomerization and fibrillization during the NMR process. Aβ residues 16–22 have been shown to participate in the transition into the β-sheet secondary structure and are independently capable of forming amyloid fibrils [42]–[44]. Furthermore, this short fragment of Aβ contains the central aromatic recognition motif of the polypeptide [44].

**Figure 3:**
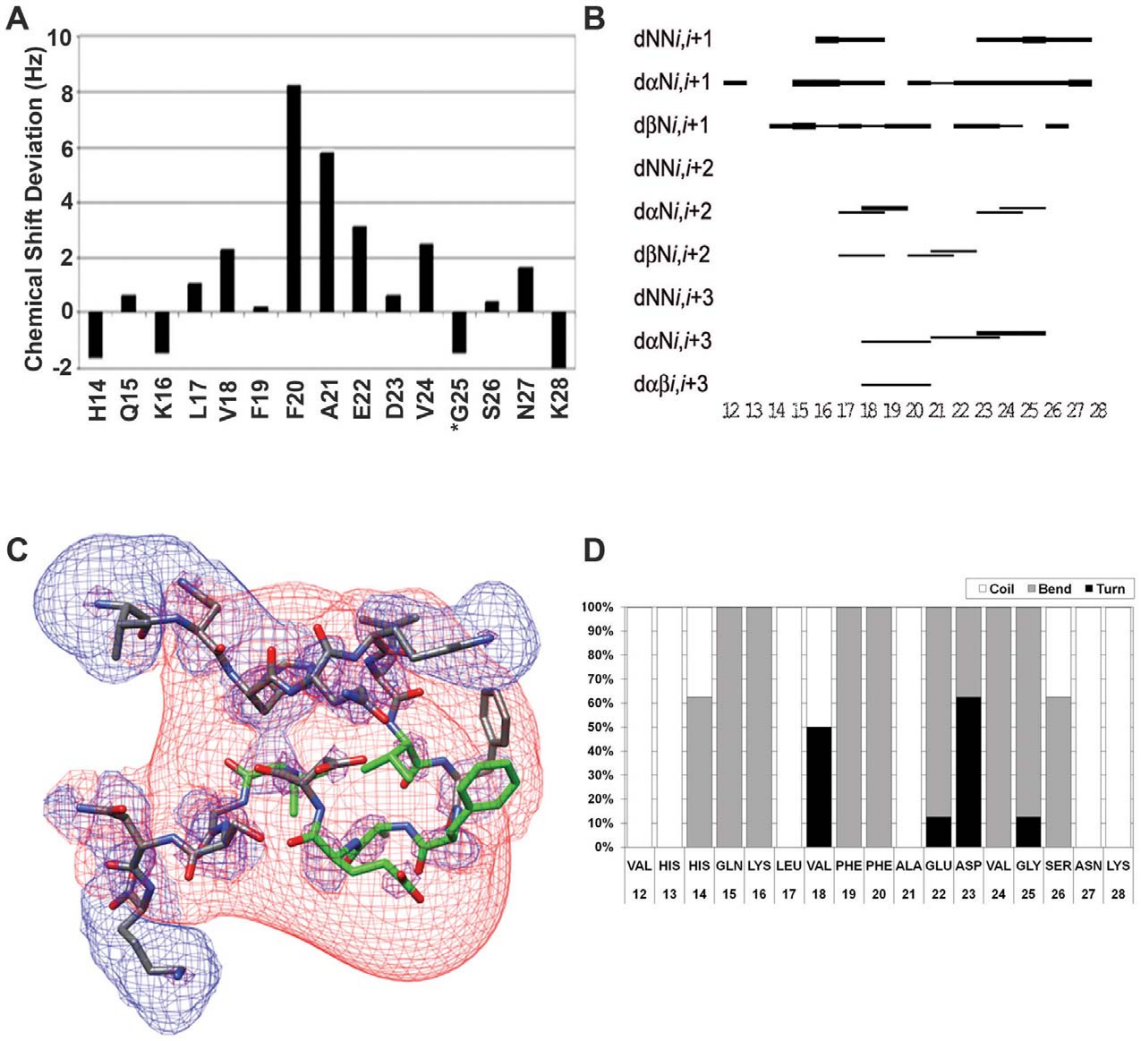
NMR analysis of Aβ with NQTrp. **A**. Amide proton chemical shifts deviations of Aβ_12–28_ residues upon interacting with NQTrp at molar ratio between 1∶0.1 and 1∶0.5 (Aβ_12–28_∶NQTrp). *Residues Lys16 and Gly25 were unresolved. **B**. NOE connectivity plot: NOE interactions are proportional to the thickness of the interconnecting lines. **C**. Lowest energy structure generated for Aβ_12–28_ with NQTrp (at 4∶1 molar ratio). Ensemble of 28 from 50 starting structures had a RMSD of 2.28 Å overall and 0.71 Å and 0.74 Å in regions 16–20 and 22–26. Residues that showed significant deviations upon binding NQTrp are colored in green. The positive (blue) and negative (red) electrostatic potential distribution for ±2 kT/e is mapped onto the structure. **D**. Secondary structure statistics: percentage of low energy structures in turn (black), bend (grey) or coil (white), secondary structures.

NQTrp was titrated into Aβ_12–28_ sample in 10 µL aliquots, in the same solvent batch as the peptide samples, to achieve increments of 0.11 mM of NQTrp per aliquot. After each addition, the ^1^H-NMR spectrum was taken. The addition of NQTrp to Aβ_12–28_ in solution affected the backbone amide chemical shifts of the peptide (Figure 3A). Changes in chemical shift at a 2∶1 molar ratio (Aβ_12–28_∶NQTrp) were compared to the average change in chemical shift of 0.1 Hz when a 0.1 mM aliquot of NQTrp was added as a control. These were most evident in residues Phe20, Ala21 and Glu22, which showed changes of 8, 5 and 3 Hz, respectively. Both Val18 and Val24, also showed a lesser change in chemical shift of 2 Hz. Non-terminal residues that were unaffected by the addition of NQTrp showed mostly chemical shift deviations of less than 1 Hz. The NMR experiments of NQTrp-Aβ_12–28_ binding thus showed the most prominent interactions in the region of Phe20 to Glu22. The changes in chemical shift indicate altered chemical environment either due to a direct interaction with NQTrp itself or due to a structural change that occurs upon binding.

The structure of Aβ_12–28_ was solved in the presence of 0.25 molar ratio of NQTrp to Aβ (Table 1 and Figure 3B). The spectrum was resolved and showed numerous interactions (Table 2, Table S2, Figure S2 and S3). Of the 50 calculated structures (RMSD 2.37 Å on the backbone), 28 had no violations and a RMSD value of 2.28 Å and 9 low-energy structures were chosen for structural analysis. (Figure S3, backbone (bb) RMSD 1.12 Å). These had three regions of stability (Figure 3D): Residues 14–16 (bb RMSD 0.71 Å) showed a number of NOE interactions between the region of His13 and His14 and Leu17; residues18–20 showed a turn including phenylalanines 19 and 20 (bb RMSD 0.12 Å). The general structure of the ensemble showed a loose β-hairpin with a turn at residues18–20 including phenylalanines 19 and 20 (bb RMSD 0.12 Å). Additional regions of stability (Figure 3D) included residues 14–16 (bb RMSD 0.71 Å) that showed a number of NOE interactions between the region of His13 and His14 and Leu17; and a turn at residues 22–26 (bb RMSD 0.0.67 Å) that were stabilized by hydrogen bonding between the amide proton of Ser26 and the backbone oxygen of Asp23 in the majority of the conformations. This turn was unexpected and may either be an artifact of working with a truncated peptide, or part of the mechanism by which NQTrp disrupts plaque accumulation.

**Table 1:** ^1^H chemical shift assignment of Aβ_12-28_.

HN	Hα	Hβ	Others	
V12		3.72	2.10	CH_3_ γ 0.89
H13	8.89	4.66	3.15	Hδ2 7.24, Hε1 8.55
H14	8.73	4.66	3.18, 3.06	Hδ1 8.49, Hδ2 7.26, Hε1 8.56
Q15	8.59	4.28	2.02, 1.94	CH_2_ γ 2.32, Hε 7.6, 6.95
K16	8.48	4.24	1.76, 1.71	CH_2_ γ 1.41, 1.34, CH_2_ δ 1.64, CH_2_ ε 2.93
L17	8.32	4.31	1.56, 1.42	CH γ 1.56, CH_3_ δ 0.89, 0.82
V18	7.96	4.01	1.88	CH_3_ γ 0.79, 0.72
F19	8.21	4.54	2.96, 2.85	CH_2_ δ 7.30, Hε7.28, Hζ7.14
F20	8.14	4.54	3.09, 2.93	CH_2_ δ 7.33, Hε7.31, Hζ7.22
A21	8.26	4.19	1.33	
E22	8.28	4.24	2.04, 1.91	CH_2_ γ 2.35
D23	8.39	4.65	2.78, 2.67	
V24	8.07	4.11	2.14	CH_3_ γ 0.92, 0.91
G25	8.50	3.94		
S26	8.13	4.42	3.84	
N27	8.45	4.70	2.80, 2.73	CH_2_ δ 7.61
K28	7.87	4.13	1.80, 1.67	CH_2_ γ 1.35, CH_2_ δ 1.62, CH_2_ ε 2.96, Hζ2 7.54
NQTrp	7.04	4.23	3.51, 3.26	CH_2_ δ 7.26, Hε1 10.18, Hε3 7.63, Hη1 7.13,

**Table 2:** NOE interaction statistics.

Total number of restraints 177
Intra-residual restraints 52
*i*+1 restraints 74
*i*+2 restraints 25
*i*+3 restraints 18
Long range restraints 8

Figure 3C shows the lowest calculated energy conformation with residues Val18, Phe20, Ala21, Glu22 and Val24, colored in green to indicate residues whose chemical shift changed upon interacting with NQTrp. The positive (blue) and negative (red) electrostatic potential distribution for ±2 kT/e is mapped onto the structure; showing the positively charged N-terminus and Lys28, and the negative potential in the central region of the Aβ12–28 peptide.

### CD characterization of the interaction of NQTrp with Aβ

**Figure 4:**
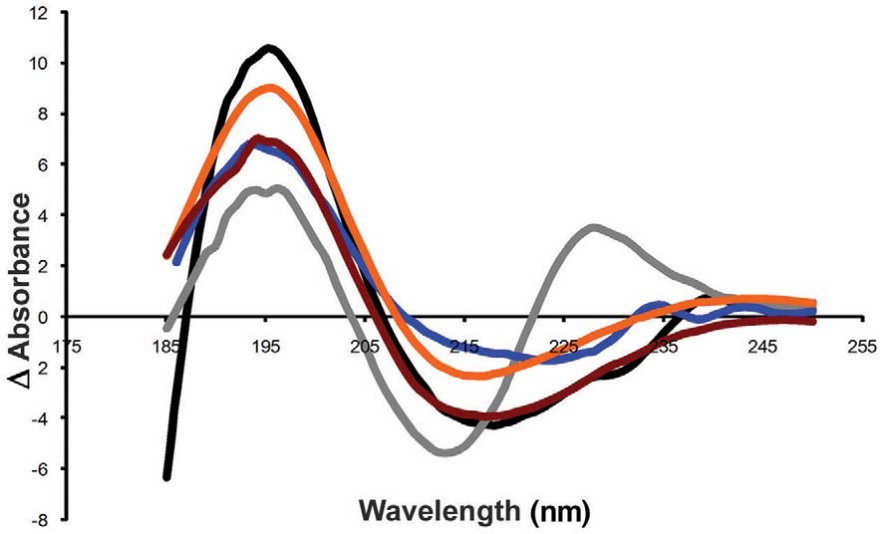
CD studies of Aβ with NQTrp. CD spectrum of Aβ_1–42_. Concentration indicated as Aβ∶NQTrp molar ratio. Control - Aβ_1–42_ only (black), 1∶60 (grey), 1∶30 (blue), 1∶1 (orange), 5∶1 (red).

Samples containing Aβ_1–42_ and NQTrp were subsequently analyzed by Circular Dichroism (CD) to gain information on the secondary structural changes that the early Aβ species undergo when incubated with NQTrp. Native Aβ_1–42_ oligomers exhibit a strong positive band around 195 nm and a negative band at 217 nm, indicating a β-sheet conformation. A dose dependent decrease in both of these bands and a small shift in the spectrum were evident with increasing concentrations of NQTrp, yet the typical β-sheet spectrum is still apparent (Figure 4). This implies that, when incubated with NQTrp, Aβ retains its β-sheet conformation, yet this conformation is gradually lost with increasing concentrations of the naphthoquinone.

### Simulation of Aβ assembly with and without NQTrp

Computer simulations were carried out to further investigate the interactions between NQTrp and Aβ. We examined the influence of NQTrp on the early phase of ordered aggregation of the central region of the Aβ peptide, focusing on the segment 14–24, centered on Phe 19 and Phe 20. A divide-and-conquer approach [46] has been adopted to efficiently sample the conformational transitions of the system. Therefore, the segment was decomposed into three overlapping heptapeptides: Aβ_14–20_, Aβ_16–22_, and Aβ_18–24_ (see sequences in Table S3). Implicit solvent molecular dynamics (MD) simulations were used to simulate the aggregation of three replicas of the considered peptides in presence and absence of NQTrp.

**Figure 5:**
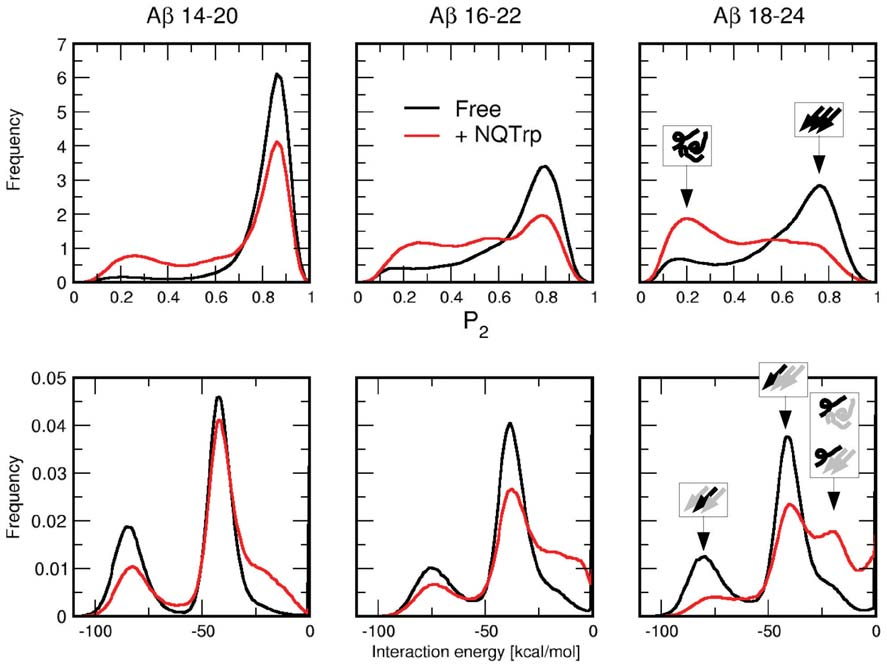
NQTrp hinders β-sheet formation. Red lines and black lines correspond to simulations with and without NQTrp, respectively. (Top) Frequency histograms of the nematic order parameter P2 for the three Aβ segments. Values of P2 close to 0.2 and 0.8 correspond to disordered conformations and β-sheet structures, respectively (see the insets in the top right plot). The presence of NQTrp sensibly increases the amount of disordered structures for all peptides. (Bottom) Inter-peptide interaction energy distributions. The two peaks of the distributions correspond to a peptide in the center of an ordered oligomer (about −80 kcal/mol) and a peptide at the edge of an ordered oligomer (about −40 kcal/mol). The shoulder of the energy distribution at values of about −20 kcal/mol contains events with disordered or partially ordered oligomers (see insets in the bottom right plot). NQTrp markedly increases the amount of structures with unfavorable inter-peptide interaction energy.

During the simulations the three-peptide system explores several configurations. The P_2_ order parameter (described in Materials and Methods) has been adopted to monitor the degree of orientational order within the oligomers: a value close to one corresponds to an ordered trimer, with either parallel or antiparallel β-sheet, while a value close to zero reflects a fully disordered system. The frequency histograms of P_2_ for the unperturbed and perturbed systems (Figure 5) display a prominent peak at P_2_ = 0.8, and a shoulder for P_2_ values lower than 0.5, which includes disordered aggregates and isolated peptides. The threshold value P_2_^*^ = 0.665 is chosen as the crossover between ordered and disordered states (see Materials and Methods) [39]. The ratio between order and disorder clearly shows that NQTrp perturbs the order of the aggregate (Table S3) by increasing the population of disordered conformations for all three peptides. The frequency distribution of inter-peptide interaction energies (Figure 5) shows two peaks. The peak at −80 kcal/mol and the peak at −40 kcal/mol correspond to a peptide interacting with the center and at the edge of an ordered trimer, respectively. From the plots it is evident that the presence of NQTrp increases the number of events with interaction energy close to zero, originating from unstructured peptides bound to the oligomeric or isolated Aβ species. The presence of NQTrp alters the number of backbone hydrogen bonds by increasing the intra-chain and decreasing the inter-chain interactions (Table S3). The simulation results indicate that the trimer structure is perturbed by NQTrp, which is able to intercalate into the oligomer and influence its structure, supporting the evidence attained above by NMR and CD spectroscopy.

### Binding mechanism of NQTrp to Aβ by computational analysis

**Figure 6:**
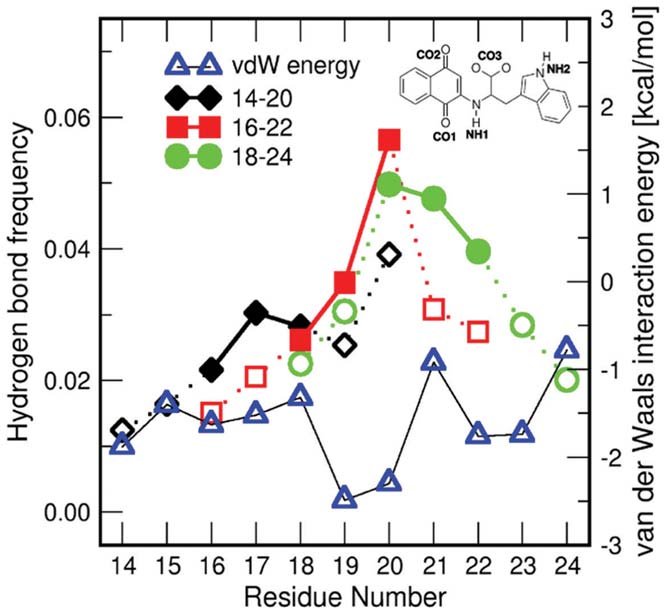
Computer analysis of the interactions between NQTrp and Aβ. Frequency of interactions between all NQTrp CO groups and peptide backbone NHs (left y-axis). Open symbols correspond to residues proximal to the N-terminal or C-terminal of the peptide (positions 1, 2, 6, and 7 in each heptapeptide). Closed symbols correspond to the central residues (positions 3, 4, and 5). Average van der Waals interaction energy between the residues and NQTrp are shown by blue triangles (right y-axis). Lower values correspond to more favorable interaction energy.

Further computational analysis was conducted in order to determine the binding mechanism of NQTrp to Aβ. Hereafter, the hydrogen bonds between NQTrp and the Aβ peptide backbone will be identified using the labels of polar groups of NQTrp (see inset of Figure 6 for the labels), e.g., NH1-CO is the hydrogen bond between NH1 group and any carbonyl group of the backbone. Furthermore the interaction with a certain residue will be specified with the amino acid name, e.g., NH1-Phe20 is the hydrogen bond between NH1 group and backbone carbonyl of Phe20, and CO1-Phe20 is the hydrogen bond between CO1 group and Phe20 backbone amide. Due to the symmetry of the carboxyl oxygens of NQTrp, the hydrogen bond that can be formed with one of the two CO moieties will be referred as to CO3-NH. The frequency of hydrogen bond formation between the carbonyl groups of NQTrp and the amide backbone is shown in Figure 6. The agreement with the NMR amide proton chemical shift deviations is remarkable. The backbone amides that interact most with NQTrp through hydrogen bonds belong to Phe20, Ala21, and Glu22. It is worth noting that, although the van der Waals interaction energies between NQTrp and Phe19 or Phe20 are very similar, there is a much higher propensity for NQTrp to form a hydrogen bond with Phe20. The most frequent hydrogen bonds involving the peptide backbone are NH1-CO, CO1-NH and CO3-NH (Figure S4, Table S4). Interestingly, the hydrogen bond pairs NH1-CO, with CO1-NH or CO3-NH occur simultaneously at high probability (about 10% of the trajectory), and very frequently the three hydrogen bonds are formed at the same time (5% of the trajectory) (Table S5). These hydrogen bonds occur either within the same residue (Phe20 or Ala21), or within two amino acids that are separated by a single residue (Val18, Phe20, or Phe20, Glu22) (Table S5).

**Figure 7:**
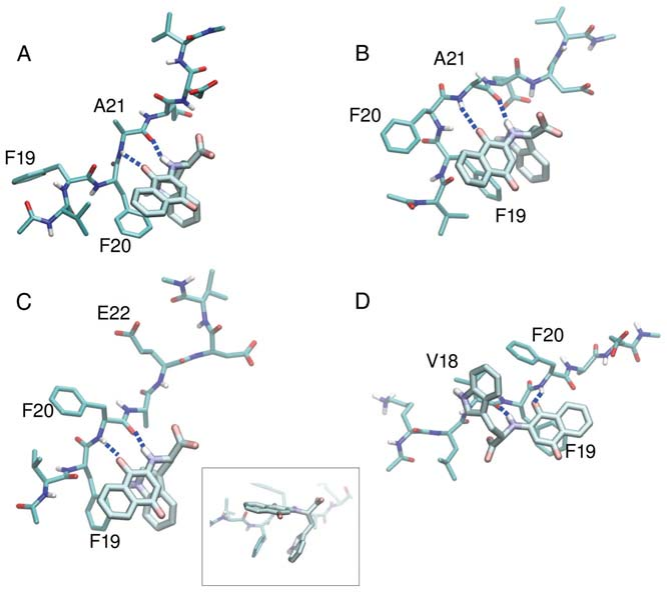
Modeling of representative snapshots of the binding modes of NQTrp to the Aβ_peptide. (**A,B**) The two most frequent conformations (12% and 9%) when NQTrp is bound to Aβ18–24 through CO1-NH and NH1-CO interactions with Ala21. The main difference between the two structures is the swap of Phe20 and Phe19 as a counterpart for aromatic interactions with NQTrp. **C** The most frequent conformation (17%) obtained when NQTrp is bound to Aβ18–24 and is involved in CO1-NH and NH1-CO interactions with Phe20. To emphasize the aromatic interactions of the naphthoquinone and the indole moieties of NQTrp with the phenyl ring of Phe19, a lateral view of the conformation **c**. is shown in the inset. **D** The most frequent conformation (11%) when NQTrp is bound to Aβ16–22 through CO1-NH with Phe20 and NH1-CO with Val18. Here the indole of NQTrp interacts with Val18, and naphthoquinone with Phe19. See inset of Fig. 5 for the labeling of the polar groups.

Notably, the MD simulations show that NQTrp strongly perturbs the ordered aggregation of the Aβ peptides by binding with specific hydrogen bonds and aromatic interactions. The snapshots shown in Figure 7 were extracted from the trajectories according to the most frequent hydrogen bond pairs (See Methods). In the most frequent binding patterns, NQTrp has a closed conformation in which the indole and the naphthoquinone “clamp” the phenyl rings of Phe19 or Phe20 (Figures 7A–C). In addition, there are stable hydrogen bonds: CO1-Ala21, and NH1-Ala21 (Figures 7A and B), or CO1-Phe20, NH1-Phe20 and CO3-Glu22 (Figure 7C). In this case Phe19 interacts with both aromatic groups of NQTrp as well. Conversely, in the presence of the NH1-Val18 and CO1-Phe20 hydrogen bonds, the indole and naphthoquinone moieties do not act as “clamp” but rather interact with the Val18 and Phe19 side chains, respectively (Figure 7D). Note that in all cases aromatic stacking and hydrogen bonds with polar groups of the backbone are present.

### NQTrp inhibits the cytotoxic effect of Aβ towards cultured cell line

**Figure 8:**
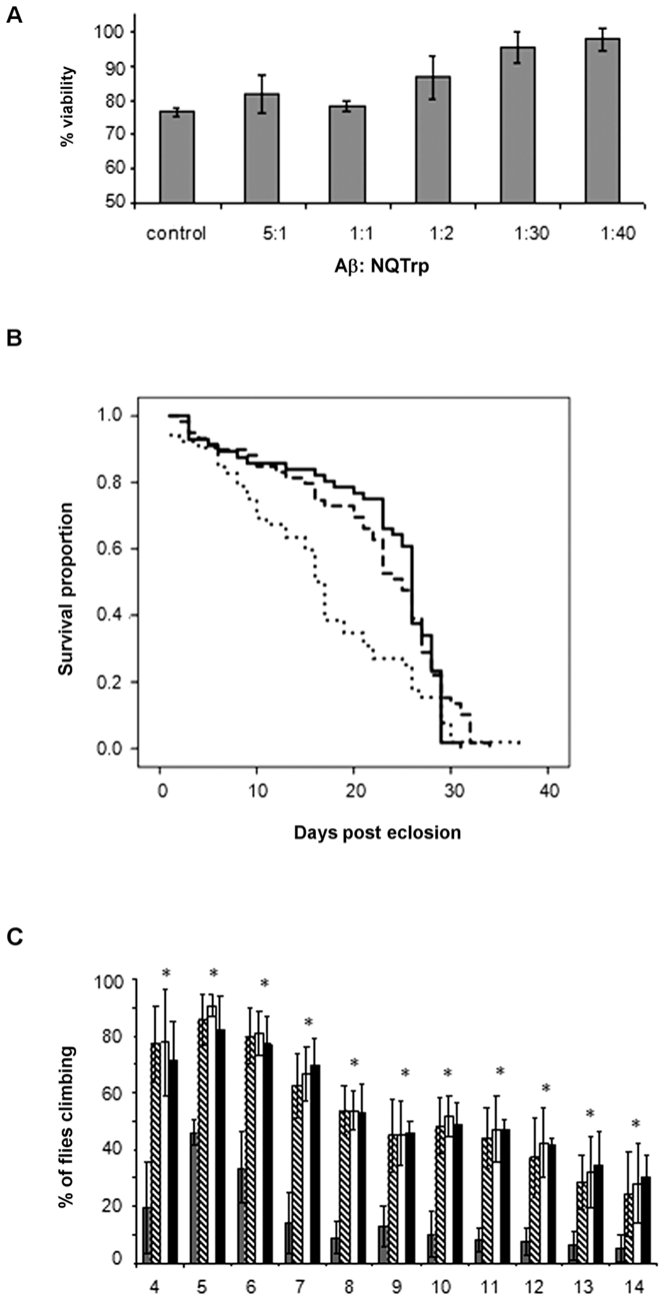
NQTrp alleviates toxic effects of Aβ – cell and fly assays. **A**. The effect of NQTrp on cytotoxicity of soluble Aβ oligmers. Soluble oligomers were prepared with and without increasing concentrations of NQTrp. The cytotoxic effect of the preparations towards cultured PC12 cells was determined using the MTT assay. Concentration are indicated as Aβ∶NQTrp molar ratio. **B**. The effect of NQTrp on longevity of Aβ_1–42_-expressing flies. The life span of four classes of flies was evaluated n = 60. Females expressing Aβ_1–42_ grown on regular medium (dotted line), females expressing Aβ_1–42_ grown on medium containing NQTrp (dashed line), males (control, carrying the Aβ_1–42_ transgene but not expressing it) grown on medium containing NQTrp males (control, carrying the Aβ_1–42_ transgene but not expressing it) grown on regular medium(not shown). **C**. The effect of NQTrp on climbing behavior of Aβ_1–42_-expressing flies.. Four classes, each containing six vials with 10 flies in each: femalesexpressing Aβ_1–42_ grown on regular medium (grey), females expressing Aβ_1–42_ grown on medium containing NQTrp (dashed line), males (control, carrying the Aβ_1–42_ transgene but not expressing it) grown on either regular medium (white) or on medium containing NQTrp (black), were analyzed using the climbing assay. Results show for each group the percent of flies climbing to the top of the vial after 18 seconds, during the course of 14 days.

To further substantiate the inhibition by NQTrp we tested whether it affects the cytotoxicity of Aβ_1–42_ oligomers towards the rat PC12 neuronal cell line. Toxic Aβ oligomers were incubated with increasing concentrations of NQTrp and cell viability was measured using the MTT assay. While showing no toxic effect of its own towards cultured cells (Figure S5), NQTrp significantly inhibited the cytotoxic effect of the Aβ oligomers and caused a significant dose dependent increase in the viability of the cells (Figure 8A). This effect was most apparent at molar excess of NQTrp which correlates with results attained from the inhibition of toxic Aβ oligomers analysis.

### The effect of NQTrp in an *in vivo* transgenic fly system

In order to assess the effect of NQTrp on Aβ in the living organism, we used a Drosophila model of AD. Transgenic flies expressing the human Aβ_1–42_ protein in their nervous system, via the Gal4-UAS system, display various symptoms reminiscent of AD including defective locomotion, and memory, which deteriorate with age, as well as markedly reduced longevity. Their brains display characteristic amyloid plaques and pathology [47].

Crossing male flies carrying the pan-neuronal elav-Gal4 driver (on their X chromosome) with females homozygous for the autosomal UAS-regulated Aβ_1–42_ transgene, resulted in female offspring expressing Aβ_1–42_ in their nervous system. The male offspring carried the Aβ_1–42_ transgene but did not express it because they lacked the Gal4 driver and served as control. This cross was performed either on regular Drosophila medium or on medium supplemented with 0.75 mg/mL NQTrp. The animals fed on the appropriate medium from the beginning of the larval stage onwards. Each class of adult offspring was monitored daily for survival and locomotion (climbing).

Flies expressing the Aβ_1–42_ transgene grown on regular medium exhibited a significantly shorter life span than the control (male) classes, as reported [47]. By day 16, only 50% of the flies expressing the Aβ_1–42_ transgene, were viable, while in the control class viability was reduced to 50% only after 26 days. The life span of Aβ_1–42_-expressing flies reared on medium containing NQTrp (Figure 8B) was much longer and was nearly identical to that of control flies grown on regular medium (50% viability observed only at day 26). The compound had no significant effect on longevity of the control flies. Statistical analysis was performed using the SPSS 15 Kaplan-Meier software package. Results show a significant difference between flies (females) expressing the Aβ_1–42_ transgene grown on regular medium versus medium supplemented with NQTrp (P<0.0005). In contrast, no significant difference was observed between Aβ_1–42_-expressing flies supplemented NQTrp and the control class grown on the same medium (P>0.8). No significant difference was seen either between the control class (males) grown on regular medium versus medium supplemented NQTrp (P>0.5) (data not shown).

Aβ_1–42_-expressing flies behaved normally at eclosion from the pupal case and subsequently developed locomotion deficits as reported [47]–[49]. At four days after eclosion these flies exhibited a marked decrease (60%) in their climbing ability becoming almost immobile by day 15, while the control classes were very active at this time (Figure 8C). In contrast, Aβ_1–42_-expressing flies reared on medium containing NQTrp displayed dramatic improvement, behaving almost identical to the control classes (males reared on medium lacking the compound) (Figure 8C). Importantly, no effect of NQTrp was observed on locomotion of the control flies. One tail ANOVA statistics showed P<0.0005 for all four classes.

**Figure 9:**
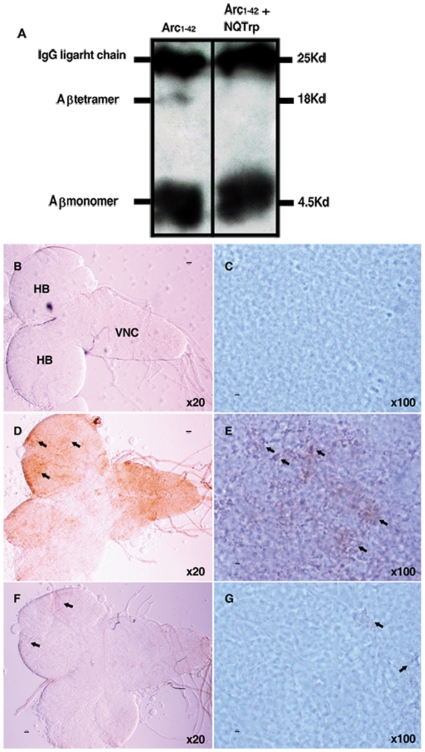
Effect of NQTrp on Aβ in larvae brains. **A**. Head extract from 6 days old Aβ_arc1-42_-expressing flies unfed (left) and fed (right) with 0.75 mg/mL NQTrp (N = 25 in each group). Accumulation of Aβ tetramers is evident only in Aβ_arc1-42_ flies which were not fed with NQTrp. (**B–G**) Immuno-staining of 3^rd^ instar larval brains with specific Aβ antibody 6E10. (**B, C**) Control animals not expressing any Aβ (elav-GAL4/+; +/+). (**D, E**) Aβ_arc1-42_-expressing animals fed with regular fly food. (**F, G**) Aβ_arc1-42_-expressing animals fed with NQTrp (elav-GAL4/+; UAS-Aβ_arc1-42_/+). N = 10 for each class examined. HB – hemi-brain; VNC – ventral nerve cord. Arrows indicate Aβ accumulation.

To further assess the curative effect of NQTrp on AD flies, Aβ was extracted from fly brains over expressing the Arctic (Arc) (E22G) mutant form of Aβ, associated with increased aggregation and early-onset familial AD [50]. These flies displayed short life span and defective locomotion as reported [47] and both of these defects were ameliorated by NQTrp as described above for Aβ_1–42_-expressing flies (data not shown). Aggregated forms of Aβ were readily detected in the soluble fraction of extracts from Aβ_arc1-42_-expressing flies following immunoprecipitation with the 6E10 Aβ-specific antibody, followed by western blot. Using this procedure monomers of Aβ were detected in head extracts of both NQTrp-fed and in non treated Aβ_arc1-42_-expressing flies. However, Aβ tetramers, which were evident in non treated Aβ_arc1-42_ flies [51], were absent from extracts of flies fed with NQTrp (Figure 9A).

**Figure 10:**
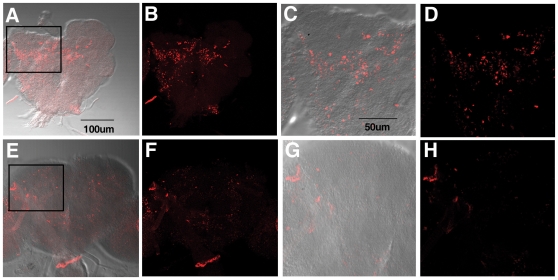
Effect of NQTrp on Aβ in drosophila brains. Immuno-staining of two-day old adult fly brains with specific Aβ antibody 6E10. (**A–D**) Aβ_arc1-42_-expressing animals fed with regular fly food (elav-GAL4/+; UAS-Aβ_arc1-42_/+). (**C, D**) Enlarged images of the boxed region. (**E–H**) Aβ_arc1-42_-expressing animals fed with NQTrp (elav-GAL4/+; UAS-Aβ_arc1-42_/+) (**G, H**) Enlarged images of the boxed region. N = 6 for each class examined.

To evaluate the effect of NQTrp on Aβ accumulation in the brains of these flies, Aβ_arc1-42_ expressing larvae and adult flies, fed or unfed with NQTrp, were immunostained with the 6E10 antibody. As reported [47], [51], both the brains of untreated larvae and adult flies displayed robust staining (Figure 9 D, E, 10 A–D) representing accumulated Aβ assemblies, not seen at all in brains of control animals not expressing any Aβ (Figure 9 A, B). Importantly, brains of Aβ_arc1-42_-expressing animals that were fed with NQTrp exhibited greatly reduced Aβ staining. (Figure 9 F, G, 10 E–H).

Taken together these results indicate that NQTrp reduced both Aβ oligomerization and accumulation in AD model flies.

## Discussion

Our work provides a rational design route toward the development of novel amyloid aggregation inhibitors of high potency. The various levels of analysis indicate that indeed the hybrid linking of naphthoquinone and tryptophan moieties leads to a highly potent inhibitor of both the oligomerization and fibrillization of Aβ with a high affinity of 90 nM and an IC_50_ of 50 nM, which is markedly lower than that reported for other aromatic Aβ inhibitors (Table S6, Supp. references S1).

Our initial hypothesis that NQTrp should interact with the central diphenylalanine recognition motif has gained direct evidence by NMR spectroscopy and *in silico* analysis. The largest chemical shift deviation was observed with Phe20 (8 Hz). A large chemical shift deviation was also observed with Ala21 and Glu22, 5 and 3 Hz, respectively. These three sequential residues form a turn in the NMR-derived conformers. The electrostatic potential of the NMR conformers suggests that peptide association may be mediated by electrostatic interactions among the distinct positive and negative regions. Interactions between the Phe19-Phe20 aromatic side chains and NQTrp may interfere with peptide-association.

This observation is further supported by the results of molecular dynamics simulations which indicate that NQTrp is involved in stable hydrogen bonds most frequently with the Phe20, Ala21 and Glu22 backbone polar groups. Remarkably, both NMR spectroscopy and computer simulations provide evidence that NQTrp binds stronger to the backbone polar groups of Phe20 than Phe19, as shown by the cluster representatives reported in Figure 7. The van der Waals interaction analysis (Figure 6) revealed favourable interaction energies between NQTrp and both the Phe19 and Phe20 side chains. In fact, when NQTrp is involved in hydrogen bonds with the backbone of the Phe20-Glu22 region, the naphthoquinone and the indole ring are able to “clamp” the phenyl ring of either Phe19 or Phe20, as shown in three of the four most frequent binding modes (Figure 7). For geometrical reasons, NQTrp does not frequently bind to the Phe19 backbone. As revealed by visual inspection of the trajectories, in this conformation NQTrp “clamps” side chain of Val18 and the resulting interaction is not favourable enough to stabilize this binding mode.

In addition to NQTrp a series of twelve quinone derivatives were screened. The main result is that a hybrid between quinone and indole is needed for optimal inhibition of both oligomerization and fibril formation. As observed in the simulations, and in agreement with the experimental inhibition assays, the presence of an electron-deficient naphthoquinone moiety, together with the electron-dense indole ring leads to the formation of a stable complex with the side chains of Phe19 and Phe20. An essential element of the active compounds (IL, IID, and III) is the presence of a three or four rotatable bonds aliphatic linker between the two aromatic moieties.

Compounds with planar aromatic rings but devoid of the aliphatic linker (molecules IV–XIII, Figure S1, Table S1) are more rigid and for this structural reason their ability of inhibiting oligomer formation is reduced.

Nevertheless, several of the molecules inactive against the oligomers are still able to inhibit the fibril formation, probably because of their ability to intercalate between the exposed side-chains [52], [53].

The main difference between IL, IID and III is the presence of a negatively charged group (only in IL, IID) which can influence the physical-chemical properties, e.g., the solubility and modify their ability of interacting with oligomers or fibrils. In addition, the most frequent hydrogen bonds with the peptide backbone of Aβ involve the quinonic carbonyls moieties, the anilinic nitrogen and the carboxyl group of NQTrp (Figure S4, Tables S4 and S5). Taken together these observations could explain the difference in activity of NQTrp and its decarboxylated analogue (molecule III, Figure S1, Table S1).

CD analysis shows a reduction in β-sheet conformation when increasing concentrations of NQTrp are titrated into the oligomeric “ordered” form of Aβ. *In silico* analysis is in accordance with these results (Figure 5). Molecular dynamics simulations revealed that NQTrp destabilizes the inter-chain backbone hydrogen bonds and increases considerably the structural disorder within the Aβ oligomer. Importantly, the inhibitory effects of the tryptophan-modified naphthoquinone on Aβ assembly *in vitro* correlate well with its effects *in vivo*. NQTrp reduced the toxicity of Aβ oligomers towards cultured cells and completely alleviated Aβ-engendered symptoms in a transgenic fly model of AD, which correlated with reduction of both Aβ oligomerization (Figure 9A) and accumulation of Aβ in the brains of these animals (Figure 9 B–G, 10 A–H).

Taken together, the results presented here for a tryptophan-modified naphthoquinone and our comparable results with D-tryptophan-Aib dipeptides [31] indicate that the targeting of the central recognition interface of Aβ by structural clamping and inhibition of further oligomerization is a promising approach for the inhibition of amyloid pathology *in vivo*. The unique properties of NQTrp and its remarkable activity *in vitro* and *in vivo* make it a promising lead for the development of small molecule inhibitors of oligomerization for the treatment of AD.

## Materials and Methods

### Compounds

1,4-naphthoquinon-2-yl-L-tryptophan (NQTrp) was synthesized from L-tryptophan and 1,4-naphthoquione by a one step synthesis according to the protocol by Shrestha-Dawadi *et al.* [39]. ^1^H-NMR (DMSO-*d_6_*): δ = 3.3 (m, CH_2_), 3.9 (m, CH_2_), 5.6 (s,1H), 6.8 (t, *J* = 3.3Hz, 1H), 6.8 (t, *J* = 7.4Hz,1H), 7.1 (s, 1H), 7.2 (br m, NH), 7.3 (d, *J* = 8.0 Hz, 1H), 7.4 (d, J = 7.5 Hz, 1H), 7.6–7.9 (m, 4H), 10.8 (NH). Reverse phase HPLC showed >95% purity. Synthetic Aβ_1–42_, Aβ_1–40_ and Aβ_12–28_ were purchased from Bachem, (Bubendorf, Switzerland).

### Determination of soluble oligomer formation

Aβ intermediates and toxic oligomers were produced according to Barghorn and coworkers [15]. To avoid pre-aggregation, synthetic lyophilized Aβ_1–42_ was pretreated with HFIP. Aβ_1–42_ was dissolved in 100% HFIP, sonicated for 20 seconds and incubated for 2 hours at 37°C under shaking at 100 RPM. NQTrp was dissolved in DMSO to a concentration of 30 mM, sonicated for 1 min and then diluted with DMSO to its final concentrations. After evaporation in a speedVac, Aβ_1–42_ was resuspended in DMSO (with or without NQTrp) to 5 mM and diluted with 20 mM NaH_2_PO_4_, 140 mM NaCl, pH 7.4 to a final concentration of 400 µM and 1/10 volume 2% SDS (final concentration of 0.2%). The toxic Aβ oligomers were generated by further dilution with two volumes of H_2_O and incubated for additional 18 hours or more (for the toxic oligomer stability assay). Aβ aggregation products were then separated using a 15% tris-tricine gel and stained using Imperial protein stain.

### Fluorescence anisotropy studies

NQTrp was dissolved in DMSO to a concentration of 50 nM and sonicated for 5 min. The solution was immediately mixed with aliquots of an Aβ_1–42_ intermediate (as described above) stock solution (20 µM) to varying final polypeptide concentrations. NQTrp polarization measurements were carried out using an ISS K2 fluorimeter. The solutions were excited at 280 nm and emission was monitored at 350 nm. For each single point, at least five measurements were collected and their average values were used for the calculation. All experiments were performed in phosphate-buffered saline, PBS [100 mM NaCl (pH 7.4)].

### ThT kinetic binding fluorescence

Synthetic lyophilized Aβ_1–40_ was dissolved in DMSO to a concentration of 100 µM and sonicated for 1 min to prevent pre-aggregation. Aβ solutions were prepared by immediate dilution with 10 mM PBS [100 mM NaCl and 0.5 mM EDTA (pH 7.4)] to a final concentration of 10 µM [containing 10% (v/v) DMSO]. The samples were diluted again to a final concentration of 5µM with the appropriate inhibitor concentration or with PBS for control samples. The samples were incubated at 37°C, and the rate of fibril formation was monitored using ThT fluorescence analysis over the course of 270 hours. The respective excitation and emission wavelengths were 450 nm (2.5 nm slit) and 480 nm (5 nm slit), respectively. A 10-fold diluted sample was taken and mixed with 900 mL of 0.4 µM ThT. The fluorescence of ThT was measured using a Jobin Yvon Horiba Fluoromax 3 fluorimeter. Each experiment was repeated in quadruplicates.

### IC_50_ ThT measurements

Synthetic lyophilized Aβ_1–42_ was dissolved in DMSO to a concentration of 100 µM and sonicated for 1 min to prevent pre-aggregation. Aβ solutions were prepared by immediate dilution with 10 mM PBS. The samples were again diluted to a final concentration of 5 µM with the appropriate inhibitor concentration or with PBS for control samples. ThT fluorescence was measured after 24 hours. The respective excitation and emission wavelengths were 450 nm (2.5 nm slit) and 480 nm (5 nm slit). A 10-fold diluted sample was taken and mixed with 900 mL of 0.4 µM ThT. The fluorescence of ThT was measured using a Jobin Yvon Horiba Fluoromax 3 fluorimeter. Each experiment was repeated in quadruplicates.

### Transmission electron microscopy

Samples of Aβ were taken after 7 days and at the end of the ThT kinetic experiment and placed on a 400 mesh copper grid covered by carbon-stabilized Formvar film (SPI Supplies, West Chester, PA). The sample was allowed to stand for 1.5 min, excess fluid was removed and the grids were negatively stained for 2 min with 10 µL of a 2% uranyl acetate solution. Excess fluid was removed, and the samples were viewed using a JEOL 1200EX electron microscope operating at 80 kV.

### NMR Analysis

#### Sample preparation

1.06 mg of lyophilized Aβ_12–28_ [was dissolved in d6-DMSO to which TDW with 0.02% w/v NaN3 was added to obtain a final sample of 1.13 mM peptide in 20% d6-DMSO solution. The order of dissolving the peptide is essential to achieve solubility.

#### NMR measurement

NQTrp was titrated into the Aβ_12–28_ sample in 10 µL aliquots in the same solvent batch as the peptide samples to achieve increments of 0.11 mM of NQTrp concentration per aliquot. After each addition the ^1^H-NMR spectrum was taken at 600 MHz with 16 scans at 21°C. Chemical shift assignment was taken from [31]; K16 and G25 were unresolved in the one-dimensional spectrum (designated by an asterisk in Fig 3A). The difference between each amide proton chemical shift and that of the peptide in the presence of with 0.1 mM NQTrp was determined for each subsequent aliquot. This value was chosen to see the effect of increasing NQTrp concentration.

Structural studies were done on the final sample from the above under the same conditions. NMR experiments were performed on a Bruker Avance 600 MHz DMX spectrometer operating at the proton frequency of 600.13 MHz, using a 5-mm selective probe equipped with a self-shielded xyz-gradient coil. The transmitter frequency was set on the hydrogen-deuterium exchange in water signal, which was calibrated at 4.811 ppm. Correlation spectroscopy (COSY) [54], total correlation spectroscopy (TOCSY), using the MLEV-17 pulse scheme for the spin lock [55], and nuclear Overhauser effect spectroscopy [56] experiments were acquired under identical conditions for all samples, using gradients for water saturation. The nuclear Overhauser effect spectroscopy experiments were acquired with a mixing time of 200 ms.

Spectra were processed and analyzed with the XWINNMR (Bruker Analytische Messtechnik GmbH) and SPARKY3 software. Resonance assignment followed the sequential assignment methodology developed by Wüthrich [57]. Stereospecificity was introduced according to the set which gave the lowest energies and RMSDs.

Electrostatic free energies were derived from finite difference solutions of the Poisson-Boltzman equation using the DelPhi program [58]. The AMBER forcefield [59] was employed and a full Coulombic calculation was performed. The positive and negative 2 kT/e isopotential surfaces were presented using [60].

### CD analysis

To avoid pre-aggregation, synthetic lyophilized Aβ_1–42_ was pretreated with HFIP. Aβ_1–42_ was dissolved in 100% HFIP, sonicated for 20 seconds and incubated for 2 hours at 37°C under shaking at 100 RPM. NQTrp was dissolved in DMSO to a concentration of 30 mM, sonicated for 1 min and then diluted with H_2_0 to its final concentrations. After evaporation in a speedVac, Aβ_1–42_ was resuspended in H_2_0 (with or without NQTrp) to 5 mM and diluted with 20 mM NaH_2_PO_4_, 140 mM NaCl, pH 7.4 to a final concentration of 400 µM and 1/10 volume 2% SDS (final concentration of 0.2%). The toxic Aβ oligomers were generated by further dilution with two volumes of H_2_O and incubated for additional 18 hours or more (for the toxic oligomer stability assay). CD measurements were conducted using quartz cuvette 0.1 mm path length, at 25°C, using AVIV 202 CD spectrometer.

### Simulation protocol and analysis

The molecular dynamics simulations were performed with the CHARMM program [61], [62]. The peptides and compound were modeled using the united atoms CHARMM PARAM19 force field with its default truncation scheme for nonbonding interactions (cutoff of 7.5 Å). Hydration effects were accounted for by using SASA, a solvent-accessible surface based implicit model [63]. Partial charges for NQTrp were computed with the modified partial equalization of orbital electronegativity algorithm (MPEOE) [64], [65]. The simulation box was prepared using the same protocol of Convertino *et al.* [39], having three monodispersed replicas of the same heptapeptide with or without the presence of a single NQTrp molecule. The concentration ratio peptide∶compound was 3∶1. Simulations were carried out with periodic boundary conditions at fixed peptide concentration of 5 mg/ml (the simulation box side was set to 98, 96 and 95 Å for Aβ_14–20_, Aβ_16–22_, and Aβ_18–24_, respectively), using Langevin integrator at low friction constant (0.15 ps) and at a temperature of 330 K, which yields reversible aggregation within a reasonable computational time. For each system, ten indipendent MD runs out of 2.5 µs each were carried out using different random number generators for the assignment of the velocities. A 2.5 µs run takes three weeks on a single AMD Opteron 252 CPU at 2.6 GHz.

Order parameters are useful quantities to monitor the structural transition within peptide oligomers [46]. In particular, the nematic order parameter allows one to measure the amount of ordered β-structure in the system:
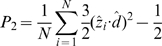
The unit vector 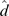, that defines a preferential direction, is the eigenvector of the order matrix that corresponds to the largest positive eigenvalue. The *N* molecular unit vectors 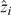 are built joining the Cα atom of residue *i* to the Cα atom of residue *i*+2 (*N* = 3×7). The values, ranging from zero to one, correspond to complete disorder and complete order respectively. The complete order is achieved when all the unit vectors are parallel or antiparallel, while the disorder is obtained when none of unit vectors is parallel to any of the others.

The threshold P_2_^*^ is a value of the order parameter chosen such that it separates the ordered from the disordered phase, and was chosen as P_2_^*^ = 0.665 [39]. Thus, the order-disorder ratio *r* is defined by the number of events where the system has a nematic order parameter lower than P_2_^*^ (disorder) and greater that P_2_^*^(order):
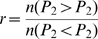
(1)Furthermore, the interference of NQTrp is measured by calculating the inter-peptide interaction energy, which is the CHARMM non-bond energy (van der Waals plus electrostatics) of a given peptide with the other two, without considering the interactions with NQTrp (Figure 5). The van der Waals interactions between NQTrp and individual Aβ residues (Figure 6) are estimated by averaging over all trajectories and neglecting the snapshots in which the interaction with all residues is zero. The criteria for hydrogen bond are the H-O distance smaller than 2.5 Å and a NH-O angle larger than 130 degrees.

Correlation between hydrogen bond pairs is calculated using the following formula:
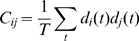
where *i* and *j* are hydrogen bond indexes, *T* is the total number of frames in the simulation, and is one when the hydrogen bond *i* is formed at time *t*, and zero otherwise.

The binding modes depicted in Figure 6 were determined by selecting the simultaneous and most frequent hydrogen bonds between the peptide backbone and NQTrp (see Table S4). Single peptide conformations that interact with NQTrp through the selected hydrogen bonds were extracted. Resulting snapshots were clustered by using an algorithm from Dr. M. Schäfer (Michael Schäfer, Syngenta Crop Protection AG, unpublished work) with a cutoff of 1.5 Å and selecting peptide heavy atoms close to NQTrp and excluding symmetrical atoms.

### Cell cytotoxicity assays

PC12 neuronal cells (2×10^5^ cells/mL) were cultured in 96-well micro plates (100 µL/well) and incubated overnight at 37°C. To each well we added 100 µL of 5 µM Aβ toxic oligomers and inhibitors at various concentrations. Each experiment was repeated four times. Following incubation for 24 hours at 37°C, cell viability was evaluated using the MTT assay. Briefly, 20 µL of 5 mg/mL MTT dissolved in PBS were added to each well. After 4 hours of incubation at 37°C, 100 µL of extraction buffer [20% SDS dissolved in a solution of 50% dimethylformamide and 50% DDW (pH 4.7)] were added to each well, and the plates were incubated again overnight at 37°C. Finally, color intensity was measured using an ELISA reader at 570 nm.

### Fly keeping

Flies were reared on standard corneal-molasses medium and were kept at 25°C. As Drosophila females can store sperm cells in their bodies, crosses were conducted using virgin females collected no longer than 8 hours after eclosion at 25°C or 18 hours after eclosion at 18°C. Adult offspring (F1) from the crosses were collected up to 9 days after the beginning of their eclosion at 25°C in order to avoid offspring from the next generation (F2).

### Fly crossing

Male flies carrying the driver elav^c155^-Gal4 (on their X chromosome) were crossed to females carrying the Aβ_1–42_ transgene (located on an autosome) under the UAS promoter in a homozygous condition. This resulted in first generation (F1) female offspring expressing Aβ_1–42_ in their nervous system. They served as our Alzheimer's Drosophila model. Male F1 offspring, which carried the Aβ_1–42_ trasgene but did not express it (because they lacked the Gal4 driver) served as a control. Animals expressing Aβ_arc1-42_ were generated in a similar way.

### Fly feeding

NQTrp was added to standard corneal-molasses medium about 10 minutes after cooking (0.75 mg/mL). The compound was mixed thoroughly into the medium and the mixture was aliquoted into rearing vials. The vials were kept at 4°C until use. Crosses were performed either on regular Drosophila medium (control) or on medium supplemented with NQTrp. Animals fed on the appropriate medium from the beginning of the larval stage onwards. Animals expressing Aβ_arc1-42_ were generated and assayed in a similar way.

### Longetivity assay

Flies expressing one copy of Aβ_1–42_ reared at 29°C on medium with and without NQTrp were classified into four classes: 1. Females expressing Aβ_1–42_, on regular medium. 2. Females expressing Aβ_1–42_, on medium supplemented with NQTrp. 3. Male controls (lacking the Gal4 driver), on regular medium. 4. Male controls (lacking the Gal4 driver), on medium supplemented with NQTrp. For each class, six vials each with 10 flies were collected and fresh food was provided every three days (whether with or without NQTrp). The number of viable Aβ-expressing and control flies treated with and without NQTrp was recorded daily post eclosion. Differences in survival rates were analyzed using the SPSS 11 Kaplan-Meir software package. Animals expressing Aβ_arc1-42_ were generated and assayed in a similar way. The longetivity assay was repeated three times. All three analyses showed similar results.

### Locomotive (climbing) assay

Test tubes of each of the four classes mentioned above, each containing 10 flies, were tapped gently on the table and were let stand for 18 seconds. The percent of flies which climbed to the top of the test tube was then calculated over time [50], [51]. Each class had six independent vial-repeats. Statistical analysis was done using StatSoft Statistica 7, parametric ANOVA testing. The locomotive assay was repeated three times. All three analysis showed similar results.

### Immuno-precipitation and western-blot of fly head extracts

Twenty five freshly decapitated heads from 6 day old Aβ_arc1-42_ flies treated and non-treated with NQTrp were collected and homogenized in 30 µl PBS/protease inhibitor/ 1% SDS following [46]. Homogenates were then centrifuged at 13000 rpm for 25 seconds and the supernatant was further immuno-precipitated with specific 6E10 anti-Aβ antibody (1∶10) over night at 4°C. Boiled samples were then western blotted and membranes were boiled in PBS for 10 minutes before antibodies were introduced. Total protein levels of the samples were quantified using Bradford analysis prior to gel loading. Since samples were loaded after IP with specific 6E10 anti-Aβ antibody, no marker protein levels could be measured.

### Immuno-staining of larval brains

3^rd^ instar larvae were dissected and stained using the following antibodies: primary 6E10 antibody (1∶250) and secondary biotinylated anti-mouse antibody detected with Vecta-Stain-Elite ABC-HRP kit (Vector Laboratories). Stained larvae brains were mounted in 70% glycerol, 30% Tris pH 7.6 and viewed using bright-field microscopy (Nikon, Eclipse E600).

### Immuno-staining of adult fly brains

Two-day old adult flies were dissected and their brains were removed. Whole brains were stained using the following antibodies: primary 6E10 antibody (1∶250) and secondary anti-mouse Cy3 (1∶100). Stained whole brains were imaged using confocal microscopy (LSM 510).

## Supporting Information

Figure S1 Structure of naphthoquione-based molecules screened for inhibition of Aβ assembly. Compounds IL and IID are L and D isomers of NQTrp.
(0.04 MB DOC)

Figure S2 1H-NMR spectra. Fingerprint regions of TOCSY (greens) spectrum overlaid on NOESY (reds) spectrum of Aβ12–28 with NQTrp (4∶1 molar ratio) with assignment.
(0.23 MB DOC)

Figure S3 1H-NMR derived structures. Ensemble of nine low energy structures generated for Aβ12–28 with NQTrp (4∶1 molar ratio).
(0.08 MB DOC)

Figure S4 Hydrogen bonds frequency between NQTrp and Aβ peptide backbone: For polar group labeling refer to the inset of Figure 5.
(0.05 MB DOC)

Figure S5 Cytotoxicity analysis of NQTrp: PC12 cell line was incubated with different concentrations of NQTrp. The cytotoxic effect of NQTrp was determined using the MTT assay. Control - no NQTrp.
(0.03 MB DOC)

Table S1 Summary of Aβ inhibition by all molecules examined: Twelve naphthoquione-based molecules were analyzed for inhibition of both oligomer and fibril formation. The relative degree of inhibition is indicated. No inhibition (−), low inhibition (+), moderate inhibition (++), significant inhibition (+++).
(0.03 MB DOC)

Table S2 Neo constraints.
(0.05 MB DOC)

Table S3 Average number of hydrogen bonds: ^a^Average number of inter- and intra-peptide backbone-backbone hydrogen bonds, with (+) and without (−) NQTrp. The standard deviation is evaluated on ten independent simulations. ^b^Ratio between order and disorder events sampled in the simulations.
(0.05 MB DOC)

Table S4 Hydrogen bond correlations: Correlation among pair of hydrogen bonds between individual polar groups of NQTrp and the peptide backbone. The pairs occurring more frequently are reported in bold. The naming convention of the polar groups of NQTrp is as Fig. 6.
(0.11 MB DOC)

Table S5 Highest probability hydrogen bonds: Pairs of hydrogen bonds with the highest probability (>0.01) to be simultaneously formed. The naming convention of the polar groups of NQTrp is as Fig. 2 bottom.
(0.06 MB DOC)

Table S6 IC50 of aromatic inhibitors of Aβ.
(0.03 MB DOC)

References S1 References for table S6.
(0.02 MB DOC)
