# Supplementary figures and images for: Complete Phenotypic Recovery of an Alzheimer's Disease Model by a Quinone-Tryptophan Hybrid Aggregation Inhibitor
Source: PLoS One. 2010 Jun 14;5(6):e11101. doi: 10.1371/journal.pone.0011101 (PMC2885425; doi:10.1371/journal.pone.0011101)

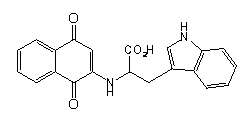

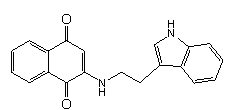

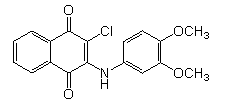

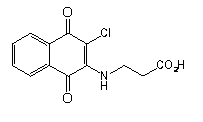

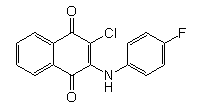

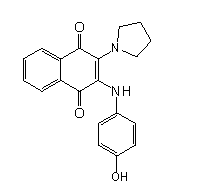

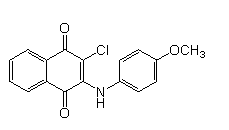

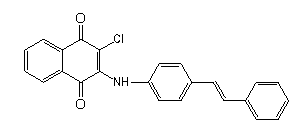

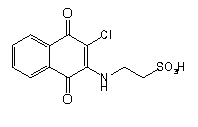

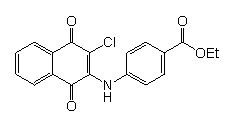

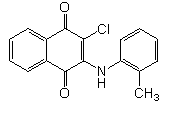


IL, IID

III

IV

V

VI

VII

VIII

VIIII

X

XI

XII

XIII


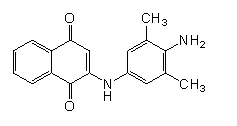


**Figure S1**

Supplement: Figure S1 — Structure of naphthoquione-based molecules screened for inhibition of Aβ assembly. Compounds IL and IID are L and D isomers of NQTrp. (0.04 MB DOC) [file pone.0011101.s001.doc]

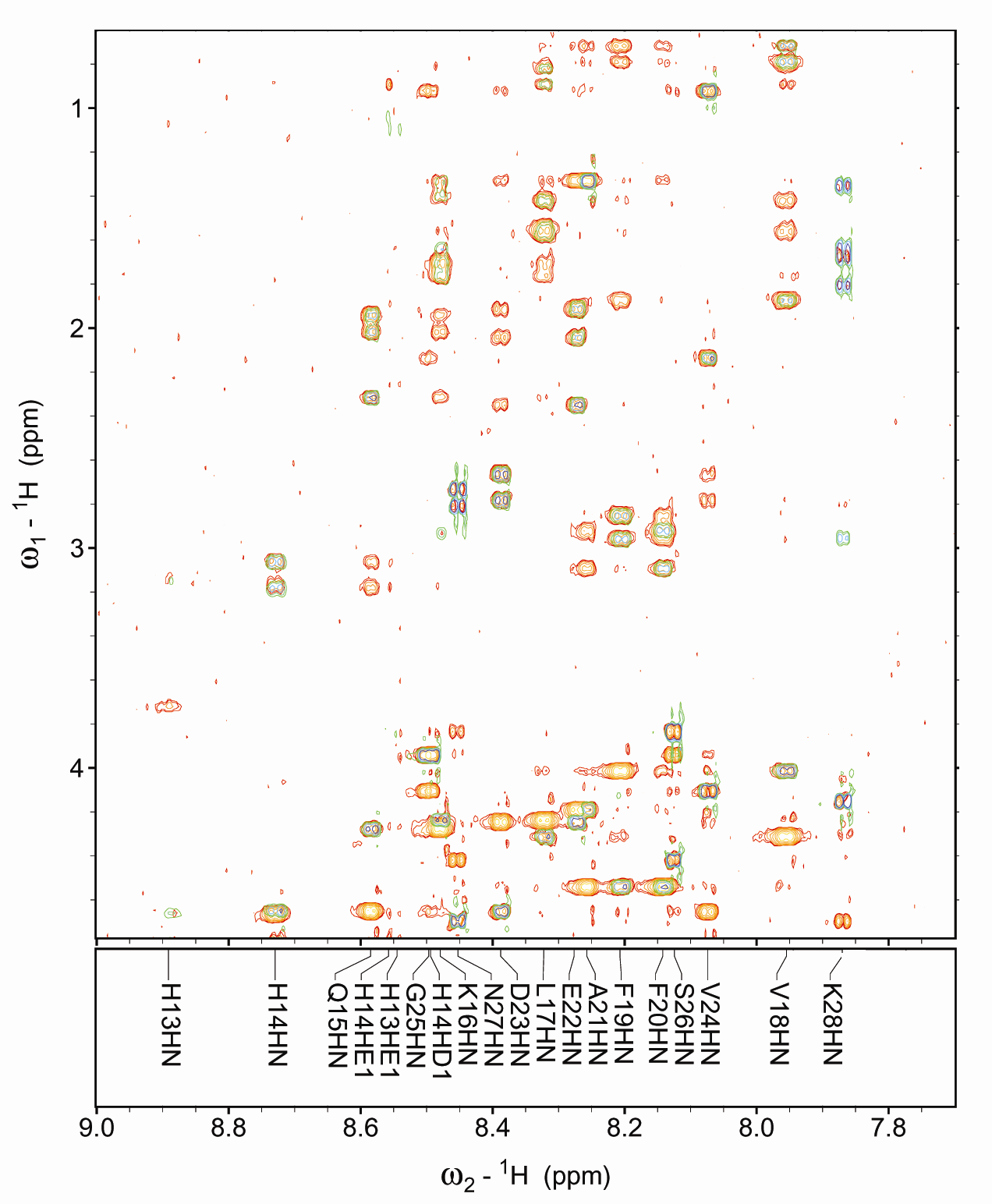


**Figure S2**

Supplement: Figure S2 — 1H-NMR spectra. Fingerprint regions of TOCSY (greens) spectrum overlaid on NOESY (reds) spectrum of Aβ12–28 with NQTrp (4∶1 molar ratio) with assignment. (0.23 MB DOC) [file pone.0011101.s002.doc]

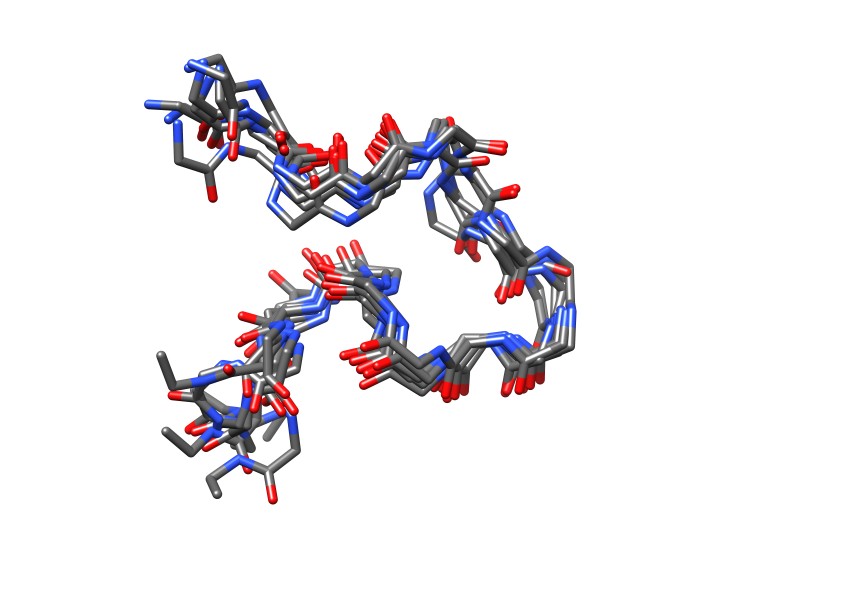


**Figure S3**

Supplement: Figure S3 — 1H-NMR derived structures. Ensemble of nine low energy structures generated for Aβ12–28 with NQTrp (4∶1 molar ratio). (0.08 MB DOC) [file pone.0011101.s003.doc]

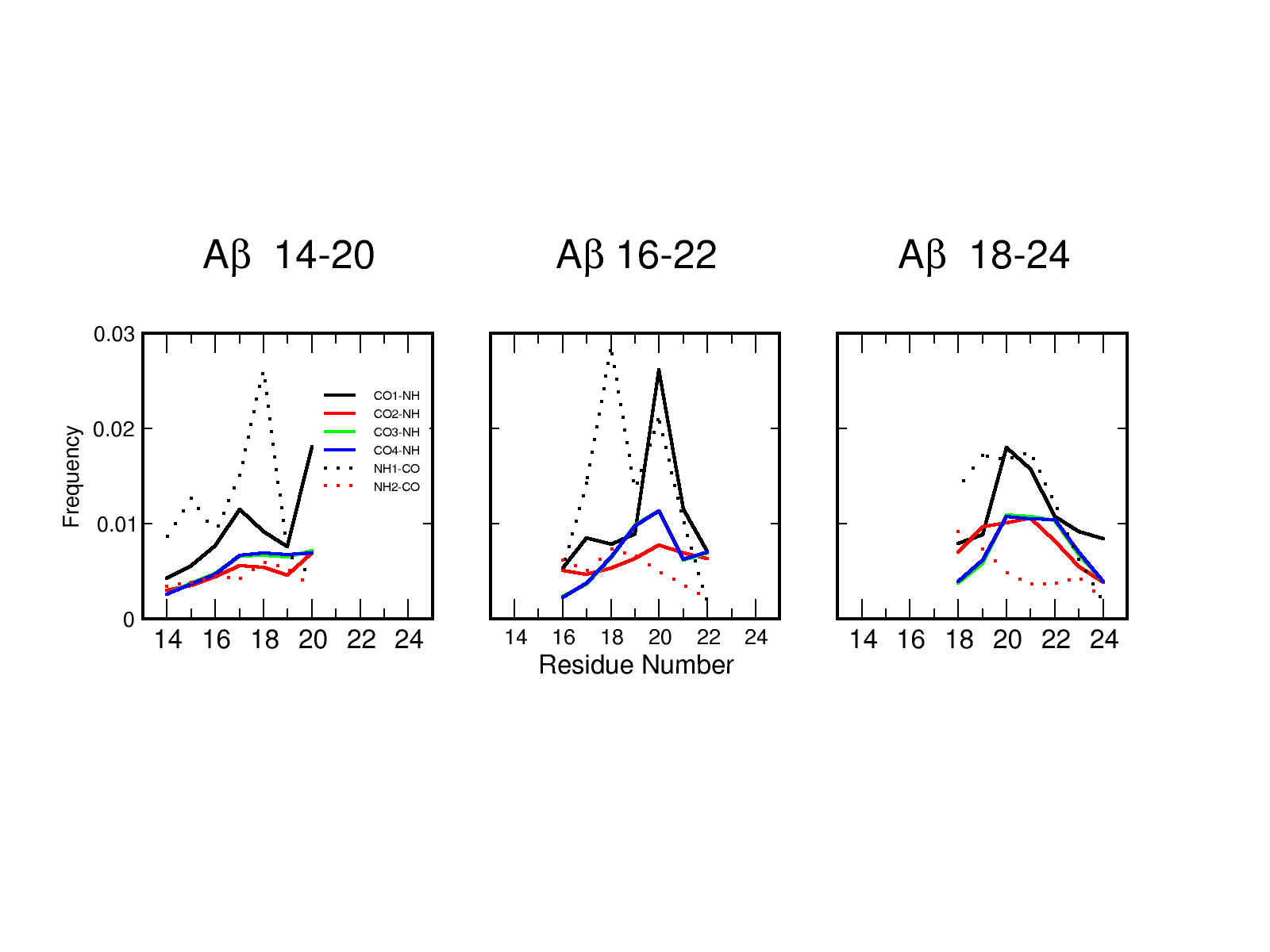


**Figure S4**

Supplement: Figure S4 — Hydrogen bonds frequency between NQTrp and Aβ peptide backbone: For polar group labeling refer to the inset of Figure 5. (0.05 MB DOC) [file pone.0011101.s004.doc]

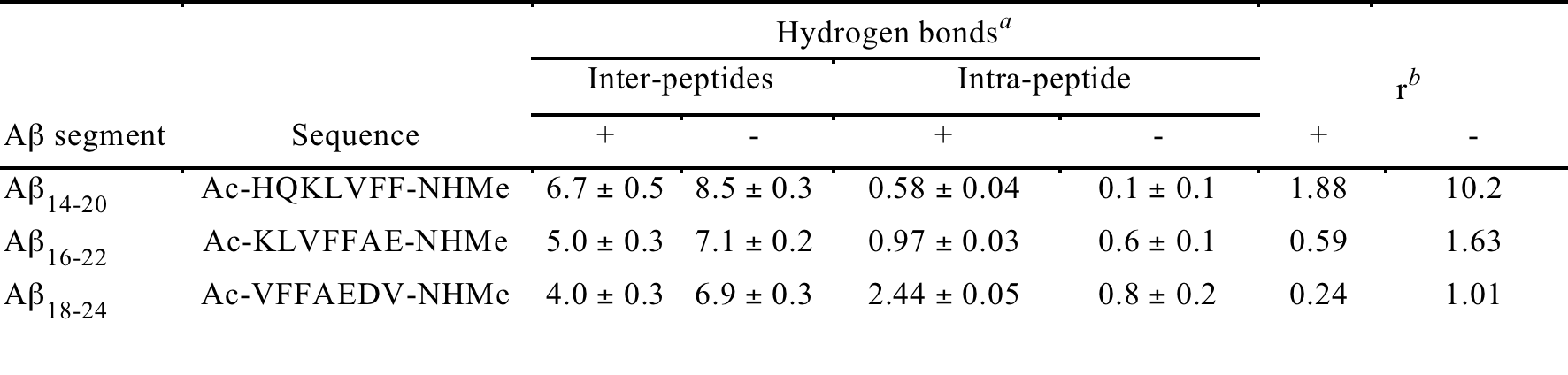


**Table S3**

Supplement: Table S3 — Average number of hydrogen bonds: aAverage number of inter- and intra-peptide backbone-backbone hydrogen bonds, with (+) and without (−) NQTrp. The standard deviation is evaluated on ten independent simulations. bRatio between order and disorder events sampled in the simulations. (0.05 MB DOC) [file pone.0011101.s008.doc]
