# Supplementary material for: Complete Phenotypic Recovery of an Alzheimer's Disease Model by a Quinone-Tryptophan Hybrid Aggregation Inhibitor
Source: PLoS One. 2010 Jun 14;5(6):e11101. doi: 10.1371/journal.pone.0011101 (PMC2885425; doi:10.1371/journal.pone.0011101)
Supplement: Figure S5 — Cytotoxicity analysis of NQTrp: PC12 cell line was incubated with different concentrations of NQTrp. The cytotoxic effect of NQTrp was determined using the MTT assay. Control - no NQTrp. (0.03 MB DOC) [file pone.0011101.s005.doc]

**Figure S5**
