# Supplementary material for: Complete Phenotypic Recovery of an Alzheimer's Disease Model by a Quinone-Tryptophan Hybrid Aggregation Inhibitor
Source: PLoS One. 2010 Jun 14;5(6):e11101. doi: 10.1371/journal.pone.0011101 (PMC2885425; doi:10.1371/journal.pone.0011101)
Supplement: Table S1 — Summary of Aβ inhibition by all molecules examined: Twelve naphthoquione-based molecules were analyzed for inhibition of both oligomer and fibril formation. The relative degree of inhibition is indicated. No inhibition (−), low inhibition (+), moderate inhibition (++), significant inhibition (+++). (0.03 MB DOC) [file pone.0011101.s006.doc]

| **Small molecule** | **Oligomer Inhibition** | **Fibril Inhibition** |
| --- | --- | --- |
| I | +++ | +++ |
| II | +++ | +++ |
| III | ++ | +++ |
| IV | - | - |
| V | - | - |
| VI | ++ | - |
| VII | - | +++ |
| VIII | + | ++ |
| VIIII | - | ++ |
| X | - | ++ |
| XI | - | + |
| XII | - | ++ |

**Table S1**
