# Supplementary material for: Complete Phenotypic Recovery of an Alzheimer's Disease Model by a Quinone-Tryptophan Hybrid Aggregation Inhibitor
Source: PLoS One. 2010 Jun 14;5(6):e11101. doi: 10.1371/journal.pone.0011101 (PMC2885425; doi:10.1371/journal.pone.0011101)
Supplement: Table S2 — Neo constraints. (0.05 MB DOC) [file pone.0011101.s007.doc]

!Abeta(12-28) with NQTrp (molar ratio 4:1, respectively)

!i.HN

4:1)

!i.HN

!!Intra-residual restraints.

!i+1.HN

!!Restraints i,i+1.

assign (resid 16 and name HN) (resid 17 and name HN) 2.3 0.5 0.5

assign (resid 18 and name HN) (resid 17 and name HN) 3.1 1.3 0.5

assign (resid 18 and name HN) (resid 19 and name HN) 3.2 1.4 0.5

assign (resid 24 and name HN) (resid 23 and name HN) 3.2 1.4 0.5

assign (resid 25 and name HN) (resid 24 and name HN) 2.9 1.1 0.5

assign (resid 26 and name HN) (resid 25 and name HN) 2.1 0.3 0.5

assign (resid 26 and name HN) (resid 27 and name HN) 3.2 1.4 0.5

assign (resid 28 and name HN) (resid 27 and name HN) 2.8 1.0 0.5

!i.FP

!!Intra-residual restraints.

assign (resid 13 and name HA) (resid 13 and name HN) 5.8 4.0 0.5

assign (resid 13 and name HB*) (resid 13 and name HN) 2.3 0.5 0.5

assign (resid 14 and name HA) (resid 14 and name HN) 2.8 1.0 0.5

assign (resid 14 and name HB2) (resid 14 and name HN) 3.1 1.3 0.5

assign (resid 14 and name HB1) (resid 14 and name HN) 3.0 1.2 0.5

assign (resid 14 and name HD2) (resid 14 and name HN) 5.2 3.4 2.3

assign (resid 14 and name HN) (resid 14 and name HD2) 5.4 3.6 0.5

assign (resid 15 and name HA) (resid 15 and name HN) 3.1 1.3 0.5

assign (resid 15 and name HB2) (resid 15 and name HN) 2.3 0.5 0.5

assign (resid 15 and name HB1) (resid 15 and name HN) 2.6 0.8 0.5

assign (resid 15 and name HG*) (resid 15 and name HN) 3.4 1.6 0.5

assign (resid 16 and name HB1) (resid 16 and name HN) 2.4 0.6 0.5

assign (resid 16 and name HB2) (resid 16 and name HN) 2.7 0.9 0.5

assign (resid 16 and name HE*) (resid 16 and name HN) 5.8 4.0 0.5

assign (resid 16 and name HG2) (resid 16 and name HN) 2.1 0.3 0.5

assign (resid 16 and name HG1) (resid 16 and name HN) 2.5 0.7 0.5

assign (resid 17 and name HA) (resid 17 and name HN) 3.0 1.2 0.5

assign (resid 17 and name HB1) (resid 17 and name HN) 3.0 1.2 0.5

assign (resid 17 and name HD2*) (resid 17 and name HN) 4.0 2.2 0.5

assign (resid 17 and name HD1*) (resid 17 and name HN) 3.2 1.4 0.5

assign (resid 18 and name HA) (resid 18 and name HN) 3.1 1.3 0.5

assign (resid 18 and name HB) (resid 18 and name HN) 3.0 1.2 0.5

assign (resid 18 and name HG1*) (resid 18 and name HN) 4.0 2.2 0.5

assign (resid 18 and name HG2*) (resid 18 and name HN) 5.0 3.2 0.5

assign (resid 19 and name HA) (resid 19 and name HN) 2.0 0.2 0.5

assign (resid 19 and name HB2) (resid 19 and name HN) 3.0 1.2 0.5

assign (resid 19 and name HB1) (resid 19 and name HN) 2.9 1.1 0.5

assign (resid 20 and name HA) (resid 20 and name HN) 2.6 0.8 0.5

assign (resid 20 and name HB2) (resid 20 and name HN) 3.2 1.4 0.5

assign (resid 20 and name HB1) (resid 20 and name HN) 3.5 1.7 0.5

assign (resid 21 and name HA) (resid 21 and name HN) 4.0 2.2 0.5

assign (resid 21 and name HB*) (resid 21 and name HN) 2.6 0.8 0.5

assign (resid 22 and name HA) (resid 22 and name HN) 3.5 1.7 0.5

assign (resid 22 and name HB2) (resid 22 and name HN) 3.0 1.2 0.5

assign (resid 22 and name HB1) (resid 22 and name HN) 3.2 1.4 0.5

assign (resid 22 and name HG*) (resid 22 and name HN) 3.2 1.4 0.5

assign (resid 23 and name HA) (resid 23 and name HN) 3.0 1.2 0.5

assign (resid 23 and name HB2) (resid 23 and name HN) 3.0 1.2 0.5

assign (resid 23 and name HB1) (resid 23 and name HN) 2.9 1.1 0.5

assign (resid 24 and name HA) (resid 24 and name HN) 3.1 1.3 0.5

assign (resid 24 and name HB) (resid 24 and name HN) 2.8 1.0 0.5

assign (resid 24 and name HG*) (resid 24 and name HN) 2.9 1.1 0.8

assign (resid 25 and name HA*) (resid 25 and name HN) 2.7 0.9 0.5

assign (resid 26 and name HA) (resid 26 and name HN) 2.2 0.4 0.5

assign (resid 26 and name HB*) (resid 26 and name HN) 2.8 1.0 0.5

assign (resid 27 and name HA) (resid 27 and name HN) 2.9 1.1 0.5

assign (resid 27 and name HB1) (resid 27 and name HN) 3.0 1.2 0.5

assign (resid 27 and name HB2) (resid 27 and name HN) 4.5 2.7 0.5

assign (resid 28 and name HA) (resid 28 and name HN) 2.0 0.2 0.5

assign (resid 28 and name HB2) (resid 28 and name HN) 4.6 2.8 0.5

assign (resid 28 and name HB1) (resid 28 and name HN) 4.5 2.7 0.5

assign (resid 28 and name HG*) (resid 28 and name HN) 2.4 0.6 0.5

!i+1.FP

!!Restraints i,i+1.

assign (resid 12 and name HA) (resid 13 and name HN) 3.1 1.3 0.5

assign (resid 14 and name HB2) (resid 15 and name HN) 3.5 1.7 0.5

assign (resid 14 and name HB1) (resid 15 and name HN) 3.2 1.4 0.5

assign (resid 14 and name HN) (resid 13 and name HD2) 6.4 4.6 2.3

assign (resid 15 and name HA) (resid 16 and name HN) 2.5 0.7 0.5

assign (resid 15 and name HB2) (resid 16 and name HN) 2.6 0.8 0.5

assign (resid 15 and name HB1) (resid 16 and name HN) 2.2 0.4 0.5

assign (resid 15 and name HG*) (resid 16 and name HN) 3.4 1.6 0.5

assign (resid 16 and name HB1) (resid 17 and name HN) 4.5 2.7 0.5

assign (resid 16 and name HB2) (resid 17 and name HN) 5.0 3.2 0.5

assign (resid 16 and name HG1) (resid 17 and name HN) 4.5 2.7 0.5

assign (resid 17 and name HA) (resid 18 and name HN) 2.6 0.8 0.5

assign (resid 17 and name HB1) (resid 18 and name HN) 3.1 1.3 0.5

assign (resid 17 and name HD1*) (resid 18 and name HN) 4.7 2.9 0.5

assign (resid 18 and name HA) (resid 19 and name HN) 2.6 0.8 0.5

assign (resid 18 and name HA) (resid 17 and name HN) 5.0 3.2 0.5

assign (resid 18 and name HB) (resid 19 and name HN) 4.0 2.2 0.5

assign (resid 18 and name HG1*) (resid 19 and name HN) 4.0 2.2 0.5

assign (resid 18 and name HG2*) (resid 19 and name HN) 3.5 1.7 0.5

assign (resid 19 and name HB2) (resid 20 and name HN) 3.0 1.2 0.5

assign (resid 19 and name HZ) (resid 20 and name HN) 4.5 2.7 2.3

assign (resid 20 and name HA) (resid 21 and name HN) 2.7 0.9 0.5

assign (resid 20 and name HB2) (resid 19 and name HN) 5.5 3.2 0.5

assign (resid 20 and name HB2) (resid 21 and name HN) 3.3 1.5 0.5

assign (resid 20 and name HB1) (resid 21 and name HN) 3.3 1.5 0.5

assign (resid 20 and name HN) (resid 19 and name HZ) 5.0 3.2 2.3

assign (resid 20 and name HZ) (resid 21 and name HN) 4.5 2.7 2.3

assign (resid 21 and name HA) (resid 22 and name HN) 3.5 1.7 0.5

assign (resid 21 and name HB*) (resid 20 and name HN) 4.5 2.7 0.5

assign (resid 21 and name HN) (resid 20 and name HZ) 5.0 3.2 2.3

assign (resid 22 and name HA) (resid 23 and name HN) 2.7 0.9 0.5

assign (resid 22 and name HB2) (resid 23 and name HN) 3.2 1.4 0.5

assign (resid 22 and name HB2) (resid 23 and name HN) 3.0 1.2 0.5

assign (resid 22 and name HG*) (resid 23 and name HN) 2.8 1.0 0.5

assign (resid 23 and name HA) (resid 24 and name HN) 2.8 1.0 0.5

assign (resid 23 and name HB2) (resid 24 and name HN) 2.6 0.8 0.5

assign (resid 23 and name HB1) (resid 24 and name HN) 2.6 0.8 0.5

assign (resid 24 and name HA) (resid 25 and name HN) 3.4 1.6 0.5

assign (resid 24 and name HB) (resid 25 and name HN) 4.3 2.5 0.5

assign (resid 24 and name HG*) (resid 23 and name HN) 4.8 3.0 0.8

assign (resid 24 and name HG*) (resid 25 and name HN) 3.3 1.5 0.8

assign (resid 25 and name HA*) (resid 24 and name HN) 5.5 3.7 0.5

assign (resid 25 and name HA*) (resid 26 and name HN) 2.8 1.0 0.5

assign (resid 26 and name HA) (resid 27 and name HN) 2.8 1.0 0.5

assign (resid 26 and name HB*) (resid 27 and name HN) 2.6 0.8 0.5

assign (resid 27 and name HA) (resid 28 and name HN) 2.5 0.7 0.5

!i+2.FP

!!Restraints i,i+2.

assign (resid 15 and name HG*) (resid 17 and name HN) 3.9 2.1 0.5

assign (resid 17 and name HA) (resid 19 and name HN) 5.0 3.2 0.5

assign (resid 17 and name HB1) (resid 19 and name HN) 4.8 3.0 0.5

assign (resid 18 and name HA) (resid 20 and name HN) 3.4 1.6 0.5

assign (resid 18 and name HG1*) (resid 20 and name HN) 5.1 3.3 0.5

assign (resid 18 and name HG2*) (resid 20 and name HN) 3.2 1.4 0.5

assign (resid 19 and name HZ) (resid 21 and name HN) 5.1 3.3 2.3

assign (resid 20 and name HB2) (resid 22 and name HN) 5.5 3.7 0.5

assign (resid 20 and name HB1) (resid 22 and name HN) 5.0 3.2 0.5

assign (resid 21 and name HB*) (resid 23 and name HN) 5.0 3.2 0.5

assign (resid 23 and name HA) (resid 25 and name HN) 5.5 3.7 0.5

assign (resid 24 and name HA) (resid 26 and name HN) 5.0 3.2 0.5

assign (resid 24 and name HG*) (resid 22 and name HN) 5.8 4.0 0.8

assign (resid 24 and name HG*) (resid 26 and name HN) 4.5 2.7 0.8

!i+3.FP

!!Restraints i,i+3.

assign (resid 18 and name HA) (resid 21 and name HN) 5.5 3.7 0.5

assign (resid 18 and name HG1*) (resid 21 and name HN) 5.2 3.4 0.5

assign (resid 18 and name HG2*) (resid 21 and name HN) 2.8 1.0 0.5

assign (resid 21 and name HA) (resid 24 and name HN) 5.5 3.7 0.5

assign (resid 23 and name HA) (resid 26 and name HN) 2.8 1.0 0.5

assign (resid 24 and name HG*) (resid 21 and name HN) 5.5 3.7 0.5

!i-lr.FP

!!Long range (further than i+3).

assign (resid 18 and name HA) (resid 24 and name HN) 5.0 3.2 0.5

assign (resid 18 and name HG1*) (resid 26 and name HN) 6.5 4.7 0.5

assign (resid 18 and name HG2*) (resid 22 and name HN) 5.3 3.5 0.5

assign (resid 23 and name HA) (resid 15 and name HN) 2.7 0.9 0.5

!i+1.AL

!!Restraints i,i+1.

assign (resid 13 and name HA) (resid 12 and name HG*) 4.6 2.8 0.5

assign (resid 17 and name HA) (resid 18 and name HB) 5.0 3.2 0.5

assign (resid 17 and name HA) (resid 18 and name HG1*) 4.5 2.2 0.5

assign (resid 17 and name HA) (resid 18 and name HG2*) 5.5 3.2 0.5

assign (resid 17 and name HB1) (resid 18 and name HG1*) 5.5 3.7 0.5

assign (resid 18 and name HA) (resid 17 and name HD1*) 5.5 3.7 0.5

assign (resid 18 and name HA) (resid 17 and name HD2*) 5.0 3.2 0.5

assign (resid 18 and name HA) (resid 19 and name HB2) 5.5 3.7 0.8

assign (resid 18 and name HA) (resid 19 and name HZ) 4.8 3.0 2.3

assign (resid 18 and name HG2*) (resid 19 and name HE*) 5.5 3.7 2.3

assign (resid 18 and name HG2*) (resid 19 and name HZ) 4.2 2.4 2.3

assign (resid 19 and name HB2) (resid 18 and name HG2*) 5.5 3.7 0.5

assign (resid 19 and name HB2) (resid 20 and name HZ) 5.5 3.7 0.5

assign (resid 20 and name HB2) (resid 21 and name HB*) 5.0 3.2 0.5

assign (resid 22 and name HG*) (resid 21 and name HB*) 4.8 3.0 0.5

assign (resid 23 and name HA) (resid 24 and name HG*) 4.5 3.7 0.8

assign (resid 23 and name HB1) (resid 24 and name HG*) 5.5 3.7 0.8

assign (resid 25 and name HA*) (resid 24 and name HG*) 4.5 3.7 0.8

!i+2.AL

!!Restraints i,i+2.

assign (resid 15 and name HG*) (resid 17 and name HD2*) 6.0 4.2 0.5

assign (resid 16 and name HG2) (resid 18 and name HG2*) 5.5 3.7 0.5

assign (resid 16 and name HG1) (resid 18 and name HG2*) 4.5 3.7 0.8

assign (resid 17 and name HD1*) (resid 19 and name HE*) 5.5 3.7 2.3

assign (resid 17 and name HD2*) (resid 19 and name HE*) 5.5 3.7 2.3

assign (resid 17 and name HD2*) (resid 19 and name HZ) 2.2 0.4 2.3

assign (resid 18 and name HG2*) (resid 20 and name HE*) 4.7 2.9 2.3

assign (resid 18 and name HG2*) (resid 20 and name HZ) 4.9 3.1 2.3

assign (resid 20 and name HB1) (resid 18 and name HG1*) 5.5 3.7 0.5

assign (resid 20 and name HB1) (resid 18 and name HG2*) 5.5 3.7 2.3

!i+3.AL

!!Restraints i,i+3.

assign (resid 17 and name HD1*) (resid 14 and name HD2) 5.0 3.2 2.3

assign (resid 17 and name HD1*) (resid 14 and name HE1) 5.1 3.3 2.3

assign (resid 17 and name HD2*) (resid 14 and name HD2) 5.5 3.7 2.3

assign (resid 18 and name HA) (resid 21 and name HB*) 5.0 3.2 0.5

assign (resid 18 and name HG1*) (resid 21 and name HB*) 5.0 3.2 0.5

assign (resid 18 and name HG2*) (resid 21 and name HB*) 4.5 2.7 0.5

assign (resid 21 and name HA) (resid 18 and name HG2*) 5.5 3.7 0.5

assign (resid 21 and name HA) (resid 24 and name HG**) 5.0 3.2 0.5

assign (resid 21 and name HB*) (resid 18 and name HG1*) 5.5 3.7 0.5

assign (resid 24 and name HG1*) (resid 21 and name HB*) 5.3 3.5 0.8

!i-lr.AL

!!Long range (further than i+3).

assign (resid 16 and name HG1) (resid 19 and name HE*) 5.5 3.7 0.5

assign (resid 16 and name HG1) (resid 19 and name HZ) 5.5 3.7 0.5

assign (resid 16 and name HG1) (resid 20 and name HZ) 5.5 3.7 0.5

assign (resid 17 and name HD1*) (resid 13 and name HD2) 5.5 3.7 0.5

assign (resid 18 and name HG2*) (resid 14 and name HD2) 5.6 3.8 0.5

assign (resid 19 and name HA) (resid 18 and name HG1*) 5.5 3.7 0.5

assign (resid 19 and name HA) (resid 18 and name HG2*) 5.0 3.2 0.5

assign (resid 19 and name HA) (resid 21 and name HB*) 4.5 2.7 0.5

assign (resid 22 and name HG*) (resid 18 and name HG2*) 5.5 3.7 0.5

**Table S2**
