# Supplementary material for: Complete Phenotypic Recovery of an Alzheimer's Disease Model by a Quinone-Tryptophan Hybrid Aggregation Inhibitor
Source: PLoS One. 2010 Jun 14;5(6):e11101. doi: 10.1371/journal.pone.0011101 (PMC2885425; doi:10.1371/journal.pone.0011101)
Supplement: Table S4 — Hydrogen bond correlations: Correlation among pair of hydrogen bonds between individual polar groups of NQTrp and the peptide backbone. The pairs occurring more frequently are reported in bold. The naming convention of the polar groups of NQTrp is as Fig. 6. (0.11 MB DOC) [file pone.0011101.s009.doc]

**Table S4**
