# Supplementary material for: Complete Phenotypic Recovery of an Alzheimer's Disease Model by a Quinone-Tryptophan Hybrid Aggregation Inhibitor
Source: PLoS One. 2010 Jun 14;5(6):e11101. doi: 10.1371/journal.pone.0011101 (PMC2885425; doi:10.1371/journal.pone.0011101)
Supplement: Table S5 — Highest probability hydrogen bonds: Pairs of hydrogen bonds with the highest probability (>0.01) to be simultaneously formed. The naming convention of the polar groups of NQTrp is as Fig. 2 bottom. (0.06 MB DOC) [file pone.0011101.s010.doc]

**Table S5**
