# Supplementary material for: Complete Phenotypic Recovery of an Alzheimer's Disease Model by a Quinone-Tryptophan Hybrid Aggregation Inhibitor
Source: PLoS One. 2010 Jun 14;5(6):e11101. doi: 10.1371/journal.pone.0011101 (PMC2885425; doi:10.1371/journal.pone.0011101)
Supplement: Table S6 — IC50 of aromatic inhibitors of Aβ. (0.03 MB DOC) [file pone.0011101.s011.doc]

| **Inhibitor** | **IC_50_ μM** |
| --- | --- |
| NQTrp | 0.05 |
| ThT | 122.19 [1] |
| Congo Red | 10, 1.99 [1,2] |
| Curcumin | 0.8, 0.18 [3,4] |
| Phenol Red | 426.25 [1] |
| Epiccatechin gallate | 3 [5,6] |
| Epigallocatechin gallate (green tea) | 0.18 [6] |
| THB (2,3,4-Trihydroxybenzophenone) | 3.1 [5,6] |
| 4-Hydroxy indole | 85 [7] |
| 3-Hydroxyindole | 100 [7] |
| Indole-3-carbinole | 200 [7] |
| several benzofurans | 28-85 [8] |

**Table S6**
