# Supplementary material for: Complete Phenotypic Recovery of an Alzheimer's Disease Model by a Quinone-Tryptophan Hybrid Aggregation Inhibitor
Source: PLoS One. 2010 Jun 14;5(6):e11101. doi: 10.1371/journal.pone.0011101 (PMC2885425; doi:10.1371/journal.pone.0011101)
Supplement: References S1 — References for table S6. (0.02 MB DOC) [file pone.0011101.s012.doc]

**Supplementary References:**

1. Necula M, Kayed R, Milton S and Glabe CG (2007) Small Molecule Inhibitors of Aggregation Indicate That Amyloid β Oligomerization and Fibrillization Pathways

Are Independent and Distinct. J. Biol. Chem. 282:10311-10324.

2. Gestwicki JE, Crabtree GR, Graef IA (2004) Harnessing chaperones to generate small-molecule inhibitors of amyloid βaggregation. Science 306:865-869.

3. Ono K, Hasagawa K, Naki H, Yamada M (2004) Curcumin has potent anti-amyloidogenic effects for Alzheimer’s beta-amyloid fibrils in vitro. J Neurosci Res. 75:742-750

4. Yang F, Lim GP, Begum AN, Ubeda OJ, Simmons MR et al. (2005) Curcumin inhibits formation of amyloid beta oligomers and fibrils, binds plaques and reduces amyloid in vivo. J Biol. Chem. 280:5992-5901

5. Taniguchi S, Suzuki N, Masuda M, Hisanaga S, Iwatsubo T, et al. (2005) Inhibition of heparin-induced tau filament formayion by phenothiazines, polyphenols, and porphyrins. J. Biol. Chem. 280:7614-7623.

6. Porat Y, Abramowitz A and Gazit E (2006) Inhibition of amyloid fibril formation by polyphenols: structure similarity and aromatic interactions as a common inhibition mechanism. Chem. Biol. Drug Des. 67:27-37

7. Cohen T, Frydman-Marom A, Rechter M and Gazit E (2006) Inhibition of amyloid fibril formation and cytotoxicity by hydroxyindole derivatives. Biochemistry 45:4727-4735.

8. Howlett DR, Perry AE, Godfrey F, Swatton JE, Jennings KH, et al. (1999) Inhibition of fibril formation in β-amyloid peptide by a novel series of benzofurans. Biochem J. 340:283-289.
